# Plankton community changes during the last 124 000 years in the subarctic Bering Sea derived from sedimentary ancient DNA

**DOI:** 10.1093/ismejo/wrad006

**Published:** 2024-01-10

**Authors:** Stella Z Buchwald, Ulrike Herzschuh, Dirk Nürnberg, Lars Harms, Kathleen R Stoof-Leichsenring

**Affiliations:** Polar Terrestrial Environmental Systems, Alfred Wegener Institute Helmholtz Centre for Polar and Marine Research, Potsdam D-14473, Germany; Department of Earth System Sciences, Universität Hamburg, Hamburg D-20146, Germany; Polar Terrestrial Environmental Systems, Alfred Wegener Institute Helmholtz Centre for Polar and Marine Research, Potsdam D-14473, Germany; Institute of Biochemistry and Biology, University of Potsdam, Potsdam D-14476, Germany; Institute of Environmental Sciences and Geography, University of Potsdam, Potsdam D-14476, Germany; Ocean Circulation and Climate Dynamics, GEOMAR Helmholtz Centre for Ocean Research Kiel, Kiel D-24148, Germany; Data Science Support, Alfred Wegener Institute Helmholtz Centre for Polar and Marine Research, Bremerhaven D-27568, Germany; Polar Terrestrial Environmental Systems, Alfred Wegener Institute Helmholtz Centre for Polar and Marine Research, Potsdam D-14473, Germany

**Keywords:** phytoplankton, zooplankton, sedimentary ancient DNA, paleoecology, Bering Sea, marine ecosystem, carbon cycle

## Abstract

Current global warming results in rising sea-water temperatures, and the loss of sea ice in Arctic and subarctic oceans impacts the community composition of primary producers with cascading effects on the food web and potentially on carbon export rates. This study analyzes metagenomic shotgun and diatom rbcL amplicon sequencing data from sedimentary ancient DNA of the subarctic western Bering Sea that records phyto- and zooplankton community changes over the last glacial–interglacial cycles, including the last interglacial period (Eemian). Our data show that interglacial and glacial plankton communities differ, with distinct Eemian and Holocene plankton communities. The generally warm Holocene period is dominated by picosized cyanobacteria and bacteria-feeding heterotrophic protists, while the Eemian period is dominated by eukaryotic picosized chlorophytes and *Triparmaceae*. By contrast, the glacial period is characterized by microsized phototrophic protists, including sea ice-associated diatoms in the family *Bacillariaceae* and co-occurring diatom-feeding crustaceous zooplankton. Our deep-time record of plankton community changes reveals a long-term decrease in phytoplankton cell size coeval with increasing temperatures, resembling community changes in the currently warming Bering Sea. The phytoplankton community in the warmer-than-present Eemian period is distinct from modern communities and limits the use of the Eemian as an analog for future climate scenarios. However, under enhanced future warming, the expected shift toward the dominance of small-sized phytoplankton and heterotrophic protists might result in an increased productivity, whereas the community’s potential of carbon export will be decreased, thereby weakening the subarctic Bering Sea’s function as an effective carbon sink.

## Introduction

Recent global warming is known to alter marine phytoplankton [1] and zooplankton communities [2] in northern oceans. Changing sea surface temperature (SST) and atmospheric partial CO_2_ levels are associated with changes in the composition and the size structure of the phytoplankton community [3]. Higher temperature, reduced seasonal sea ice cover, and increased CO_2_ levels have been shown to be associated with a dominance of picosized bacterioplankton (0.2–3 μm) over silicified microphytoplankton (20–200 μm) [4-7]. Although bacterioplankton might be more efficient in CO_2_ fixation at warmer temperatures [8], their lower nutritional value [9, 10] and the fast recycling in the microbial loop might reduce the local or global efficiency of the biological carbon pump and decrease effective fluxes of nutrients and energy through the food web [11].

Multiple levels in the pelagic food web are occupied by diverse zooplankton [2]. As primary consumers, zooplankton play a major role in mediating the export of photosynthetically produced biomass to the deep oceans [12]. Although larger crustaceous zooplankton are assumed to contribute extensively to the carbon transport [13], heterotrophic protists feeding on bacteria and detritus are an important component of the microbial loop [14]. Phytoplankton community size structure, macromolecular composition, and growth rate can have bottom-up effects on primary consumers [15]. For example, egg production of herbivorous zooplankton can be reduced due to lower fatty-acid content of some phytoplankton species under elevated CO_2_ concentrations [16]. By contrast, the phytoplankton community in Arctic ecosystems is assumed to be top-down controlled due to high grazing rates [17].

Studying time series of communities covering past glacial and interglacial periods provides valuable information about long-term trends in the community composition under natural conditions, improving our understanding of community responses to present and future environmental change. To date, sedimentary ancient DNA (*sed*aDNA) is the only approach to identify past biological communities at a high taxonomic breadth and resolution, including taxa that leave no diagnostic features in the fossil record. The detectability and taxonomic classification of *sed*aDNA depend on the DNA preservation in the sediment. Cool and anoxic environments, like deep sea sediments [18, 19], and the presence of sedimentary mineral particles that bind extracellular DNA are beneficial for DNA preservation. Applying *sed*aDNA amplicon sequencing is a well-implemented paleo-proxy to identify the past taxonomic composition of plant [20-22] and diatom communities [23, 24] in lake or marine sediments [25-27]. A newer *sed*aDNA approach uses shotgun metagenomics, but it has only been applied in a few marine studies so far [18, 19, 28]. This approach is not target-specific and provides a broad picture of the variety of organisms that characterize past ecosystems [18, 19]. With *sed*aDNA being preserved over thousands of years, including the last glacial–interglacial cycle, the shotgun approach offers the possibility to reveal local warm- and cold-adapted communities and their compositional changes in a changing environment.

Here, we analyze *sed*aDNA from the western Bering Sea plankton community extracted from a sediment core covering a time period from 1.82 to 124 ka BP, utilizing both the metagenomic shotgun approach and amplicon sequencing of the rbcL gene of diatoms. The shotgun approach will be used to investigate the total phytoplankton DNA preserved in the sedimentary record. Additionally, we will focus on the diatom community, which will be studied in higher taxonomic resolution up to amplicon sequence variant (ASV) level. Both the total phytoplankton community and the diatom community will be investigated also in relation to heterotrophic and crustaceous zooplankton abundance recovered from *sed*aDNA.

The Bering Sea is located at the transition between the subarctic and Arctic climate zones, and its ecosystem is particularly sensitive to climatic changes [29] due to varying sea ice cover [30] and nutrient input, and therefore, changes in light availability and primary productivity over the last glacial–interglacial cycle [31-33]. In particular, warming SST in the modern Bering Sea results in an increased primary productivity and unusually long-lasting algal blooms, e.g. >4-month long coccolithophores blooms recorded for the first time in 1997 after anomalously warm summer air temperatures [34]. Satellite time series data of variations in air-sea CO_2_ flux support that the function of the Bering Sea as a carbon sink increased during the last decade [35].

Thus, as the Bering Sea is especially exposed to modern environmental change, insights about past community composition changes could improve projections of future plankton communities and marine ecosystem functioning. *Sed*aDNA is more likely to be preserved in the cold deep-water of the subarctic Bering Sea, and with relatively high sedimentation rates of 3–30 cm/kry [32], it can be studied in a reasonable temporal resolution over geological time scales.

We hypothesize a community change toward photoautotrophic picosized bacteria and heterotrophic protists during warm interglacial periods, with the cool glacial period characterized by microphytoplankton and crustaceous zooplankton. Such shifts in community composition and size distribution of the plankton putatively affect the efficiency of the biological carbon pump and contribute to changes in the global CO_2_ cycle.

## Materials and Methods

### Coring locality

**Figure 1:**
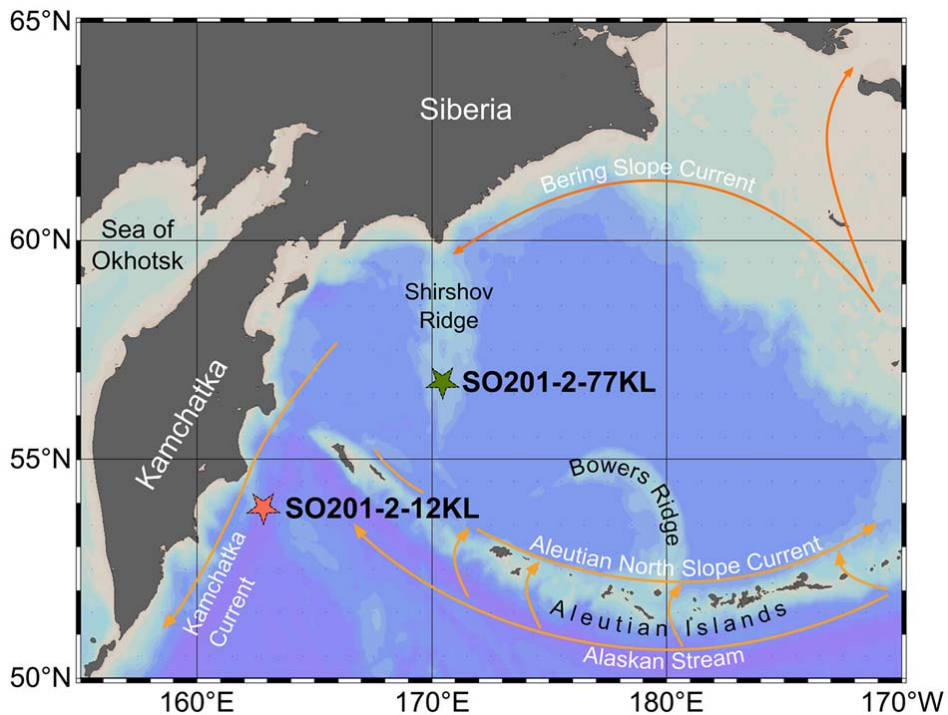
Bathymetric map of the western Bering Sea; the stars mark the sediment core SO201-2-77KL (this study) and a previously studied sediment core SO201-2-12KL [19, 25, 31, 32]; a simplified surface circulation pattern is indicated by the arrows; this map was generated with Ocean Data View [76].

During the R/V Sonne cruise SO201-KALMAR Leg 2 in 2009, the sediment core SO201-2-77KL (77KL) was recovered from the Shirshov Ridge (56.3305°N, 170.6997°E; Fig. 1) from a water depth of 2135 m [32]. The chronostratigraphical approach for sediment core SO201-2-77KL is presented in the Supplementary Information (Table S1) and in the original publications [31, 32].

Core 77KL records the last ~124 000 years (kyr), including the last interglacial, the Eemian (~130.0–119.0 ka before present (BP); [36]), the last glacial period (119.0–19.0 ka BP) including the last glacial maximum (LGM; ~26.5–19.0 ka BP; [37]), the deglaciation (~19.0–11.5 ka BP; [38]) including the rapid climatic changes to the warm Bølling–Allerød (B/A) (~14.7–12.9 ka BP) and the cool Younger Dryas (YD) climatic rebound (~12.9–11.7 ka BP; [38]), and the current interglacial, the Holocene (last ~11.7 kyr).

### Sediment sampling and sedimentary ancient DNA approaches

Core 77KL was sampled at 54 depth sections [39] with the youngest sample (5 cm) dated at about 1.82 kyr and the deepest sample (1 163 cm) at about 123.86 kyr. DNA extraction and extraction blanks (EBs) were undertaken in dedicated paleogenetic DNA laboratories at the Alfred Wegener Institute (AWI) Potsdam, Germany, using the DNeasy PowerMax Soil Kit (Qiagen, Germany). DNA purification and concentration were done with the GeneJET polymerase chain reaction (PCR) Purification Kit (Thermo Fisher Scientific). Sample DNA solutions were diluted to 3 ng/μl. For the shotgun approach, a selection of 42 DNA extracts, six associated EBs, and seven library blanks were used for library preparation as described elsewhere [40, 41]. We selected samples to cover climatic transitions (end of the Eemian and beginning of the glacial period, the deglaciation, and start of the Holocene) with higher temporal resolution than the glacial period to capture community changes during these periods of relatively rapid climate change. After indexing PCR with 10–13 PCR cycles for samples and EBs with unique combinations of P5 and P7 Illumina sequencing adapters, PCR products were purified with the MinElute PCR Purification Kit (Qiagen, Germany), and the DNA concentration was measured with Qubit 4.0 fluorometer (Invitrogen). Sample libraries were pooled equimolarly to 20 nM, while 1 μl of each extraction and library blank was added to the pool. Pooled samples were sequenced with a customized sequencing primer CL72 on two independent runs on a NextSeq 2000 System (Illumina) using paired-end mode (2 × 100 bp) with an expected output of 1 billion reads at AWI, Bremerhaven, Germany.

For the amplicon sequencing approach, we used 54 samples (diluted to 3 ng/μl) and their EBs for PCR amplifications with primers rbcl_705 and rbcl_808 [25, 42] containing a unique sequence tag of 8 bp and a random NNN suffix. PCRs were done in three replicates using 3 μl DNA and the Platinum *Taq* High Fidelity DNA Polymerase (Invitrogen). Along with each PCR batch of 10 reactions, a PCR nontemplate control was added. Replicates were pooled and purified with the MinElute PCR Purification Kit (Qiagen, Germany). DNA concentration was measured with the Qubit 4.0 fluorometer (Invitrogen). Purified PCR products were pooled equimolarly. EBs and PCR negative controls were added with 5 μl to the pool. A final MinElute was used to adjust the concentration and volume of the sample pool. Library preparation and paired-end amplicon sequencing (2 × 150 bp) on the NextSeq 500 System (Illumina) was done at Fasteris Genesupport SA sequencing service (Geneva, Switzerland) on a shared mid output flow cell with an expected output of 35 million reads. Details on sampling, DNA extraction, and shotgun and amplicon sequencing approaches are given in the Supplementary Information (Supplementary Figs S1–S7).

### Processing of DNA sequencing data

The bioinformatic analysis of raw sequences from the shotgun approach included a quality check using FastQC (version 0.11.9), deduplication of unique reads with BBmap (version 38.87), adapter trimming and merging using Fastp (version 0.20.1), and taxonomic classification of merged and paired reads with Kraken2 (version 2.1.1; [43]) at a confidence threshold of 0.2 and against the nonredundant nucleotide database (built with Kraken2 in April 2021). DNA damage pattern analysis was performed on sample groups and for selected phytoplankton and zooplankton taxa by the automated HOPS 0.34 pipeline [44] (Supplementary Figs S8 and S9). Using the R software version 4.1.2 (www.r-project.org), the classified reads were filtered for marine phototrophic families and their zooplankton consumers (including crustaceous and gelatinous zooplankton and heterotrophic protists) (Supplementary Data 1–3). Phytoplankton was grouped (e.g. phototrophic bacteria, phototrophic protists, chlorophytes) and categorized according to cell size: picophytoplankton (0.2–3 μm), mainly phototrophic bacteria; nanophytoplankton (3–20 μm), including small chlorophytes; and microphytoplankton (20–200 μm), which comprises diatoms (Sipplementary Fig. S10).

The raw paired-end sequencing data of the diatom amplicon sequencing were analyzed with the Python package OBITools 3.0.1 [45], which merges paired-end reads, demultiplexes samples, and performs taxonomic classification based on sequence similarity to the customized *rbcL-EMBL* nucleotide reference database (Supplementary Information). The resulting dataset was further filtered at the ASVs level according to similarity (96%–100%) to entries in the *rbcL-EMBL* database, taxonomic resolution, and minimum read counts. After filtering, counts of the three PCR replicates of a sample were merged (for replicate similarity see Supplementary Fig. S11). A more detailed description of the DNA data analysis is given in the Supplementary Information.

### Multivariate statistics on sedimentary ancient DNA data

The statistical analysis was performed in the R software (version 4.1.2) using the *vegan* package (version 2.5-7; [46]), and plots were produced with the *ggplot2* package (version 3.4.0). Count data of diatom amplicon sequencing samples and phyto- and zooplankton obtained from shotgun metagenomics were normalized by resampling 100 times [47]. The resampling sample size was set to the minimum read count (*N*_min_) in all samples of the respective dataset (diatom amplicon sequencing *N*_min_ = 9466, shotgun phytoplankton *N*_min_ = 984, shotgun zooplankton *N*_min_ = 64). Main taxa were defined by filtering the resampled datasets (shotgun: occurrence of a family in at least 20 samples and an occurrence in at least 1 sample with a relative abundance of ≥3%; diatom amplicon sequencing: occurrence of an ASV in at least 30 samples and an occurrence in at least 1 sample with a relative abundance of ≥3%). These filtered datasets of the main taxa represent 78.4%–91.7% of the resampled phytoplankton dataset and 70.1%–93.2% of the resampled diatom amplicon sequencing dataset. Prior to running a principal component analysis (PCA), the main families and ASVs were Hellinger transformed. A redundancy analysis (RDA) used environmental parameters as explanatory variables. Stable oxygen isotope data from the North Greenland Ice Core Project (δ^18^O NGRIP; [48]) were used as a proxy for climate variability in the Northern hemisphere. Resampled and Hellinger transformed zooplankton abundance was used as a proxy for biotic interactions. To select appropriate zooplankton families, collinearity within the zooplankton dataset was detected by calculating variance inflation factors (VIFs). Thus, a selection of three zooplankton families for which all VIFs were lower than 2 were used as explanatory variables. The family *Calanidae* (copepods) represents a group of crustaceous zooplankton, and *Apusomonadidae* and *Salpingoecidae* represent heterotrophic protists. The latter two showed moderate correlation of ρ = 0.50 (*P* < .01) in a Shapiro–Wilk test, therefore their relative abundances were summed before Hellinger transformation to represent the effect of heterotrophic protists. The total variance explained by the explanatory variables and their unique effect on the phytoplankton community composition were calculated. All *P*-values were calculated by performing an ANOVA with a significance threshold of *P* < .05.

## Results

### Temporal shifts in plankton communities in shotgun sequencing

Sequencing of 42 samples, 6 EBs, and 7 library blanks resulted in 188 405 079 classified paired and merged reads (10.39% of raw read pairs). The read pairs in all blanks sum up to 4 464 236 (0.25% of raw read pairs). After filtering, 66 phytoplankton families (120 580 reads) and 37 zooplankton families (9172 reads) were detected (Supplementary Data 1, Supplementary Fig. S10). Filtered reads for plankton families are given in Supplementary Data 2–4). Although the DNA concentration of the samples is negatively correlated with sample age, no correlation was detected between the total number of classified reads and sample age (Supplementary Data 5, Supplementary Fig. S2). The presence of damage patterns indicated by an increased number of C > T changes at the 5′-3′ end of the phyto- and zooplankton DNA reads supports the authenticity of our results (Supplementary Figs S8 and S9). Phytoplankton taxa show an increase in C > T pattern with sample age. The zooplankton reads show less strong C > T changes, probably because of the low number of DNA reads.

For the last ~124 kyr, six climatic zones are defined according to temperature reconstructions from the Northern hemisphere (see Materials and Methods). We generally categorize them into warm periods: the Eemian interglacial (5 samples), the deglacial B/A interstadial, and the Holocene interglacial (11 samples); or cold periods (in total 26 samples) of stadial-interstadial changes between ~119.0 and 26.5 ka BP, summarized as the “glacial period,” followed by the LGM and the YD.

**Figure 2:**
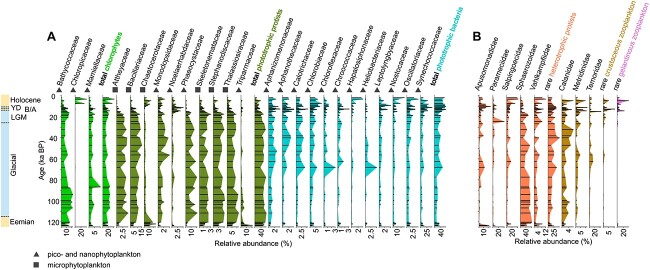
Main phytoplankton and zooplankton families plotted versus age; relative abundance is calculated from all phytoplankton (A) and zooplankton reads (B) of the respective sample after resampling; only those families that occur in at least 20 samples and in at least 1 sample with a relative abundance ≥3% are shown; the relative abundance of the remaining rare zooplankton taxa is summarized as heterotrophic protists and crustaceous and gelatinous zooplankton; warm periods (Holocene, B/A, Eemian) and cool periods (YD, LGM, and the last glacial period) are indicated on the left.

The phytoplankton communities in the samples of cold periods are dominated by microsized phototrophic protists (32.2%), mainly comprising the diatom families *Bacillariaceae* and *Thalassiosiraceae* and nanosized haptophytes within the family *Phaeocystaceae*. By contrast, picosized phototrophic bacteria (18.5%), mainly *Synechococcaceae*, and chlorophytes (11.1%), mainly *Bathycoccaceae*, are less than phototrophic protists (Fig. 2A). The phytoplankton communities in samples from all warm periods together are dominated by picosized phototrophic bacteria (18.6%), mainly the families *Synechococcaceae* and *Nostocaceae*. Phototrophic protists (12.6%), mainly *Chaetocerotaceae* and *Triparmaceae*, and chlorophytes (6.9%), mainly *Bathycoccaceae* and *Chloropicaceae*, are less abundant than phototrophic bacteria during warm periods (Fig. 2A). The composition of the warm period communities in the Holocene and B/A differs from the Eemian community composition. Pico- and nanosized phototrophic protists (56.7%) dominate in the Eemian compared to a lower relative abundance in the Holocene and B/A (22.3%). Although *Chaetocerotaceae* and *Thalassiosiraceae* are dominant during interglacials, the phototrophic protist communities in the Holocene and B/A are subdominated by *Noelaerhabdaceae*, whereas Eemian samples show higher relative abundance in *Triparmaceae*. Furthermore, phototrophic bacteria are more abundant in the Holocene and B/A (59.0%) than in the Eemian (26.2%). Picosized chlorophytes exhibit similar relative abundance, but Holocene and B/A samples are more abundant in *Chloropicaceae* (8.4%), whereas in Eemian samples *Bathycoccaceae* predominate. Further we identified plankton community shifts during the Holocene toward a relative increase of cold-adapted families, like *Phaeocystaceae* and *Bacillariaceae*, in the sample at 4.68 ka BP.

Zooplankton communities show more similar relative abundances over the last glacial–interglacial cycle compared to the phytoplankton communities, with two families of heterotrophic protists, *Sphaerozoidae* and *Salpingoecidae*, being most dominant in all samples (Fig. 2B). Among crustaceous zooplankton, the family *Temoridae* occurs in similar abundance in cool and warm periods, whereas *Calanidae* are subdominant in cool periods and *Metridinidae* dominate during warm times. Gelatinous zooplankton shows highest abundance in warm periods. Holocene and B/A samples have higher abundances of crustaceous and gelatinous zooplankton (together 27.7%) than Eemian samples (5.7%).

### Temporal shifts in diatom communities from amplicon sequencing

**Figure 3:**
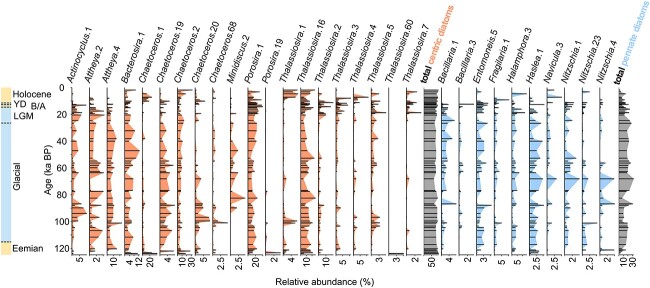
Main diatom genera inferred from amplicon sequencing of the rbcL gene of diatoms plotted versus age; relative abundance is calculated from all reads assigned to at least genus level in the respective sample within the filtered dataset after resampling; only genera that occur in at least 30 samples and in at least one sample with a relative abundance ≥3% are shown; warm periods (Holocene, B/A, Eemian) and cool periods (YD, LGM, and the last glacial period) are indicated on the left.

Amplicon sequencing of the rbcL gene of diatoms resulted in 16 143 036 reads assigned to 12 123 ASVs. Filtering resulted in 8 660 773 reads (53.7% of the raw dataset) and 475 ASVs were assigned to 35 genera within 20 diatom families (Supplementary Tabel S2). Centric diatoms dominate the samples of both cool and warm periods, but the proportion of pennate diatoms is higher in cool periods than in warm periods, while the relative abundance of total centric diatoms reveals maxima during interglacials (Fig. 3). *Thalassiosiraceae* (199 unique ASVs) and *Chaetocerotaceae* (102 unique ASVs) are the most abundant in all samples. The centric genera *Attheya* (42 unique ASVs) and *Actinocyclus* (12 unique ASVs) occur at lower relative abundance during the warm periods (1.0% and 0.3%, respectively), but these genera reach, on average, 7.7% and 2.0%, respectively, during the glacial periods. Similarly, the pennate diatom genus *Haslea* (8 unique ASVs) shows higher relative abundance in samples from the glacial period (average 1.7%, max. 5.5%) compared to interglacial samples (average 0.3%, max. 3.5%). Comparing warm period samples indicates a higher relative abundance of *Attheya* and *Haslea* in Eemian than in Holocene samples, whereas *Actinocyclus* shows an opposite trend. During the generally warm Holocene, we detected an increase of pennate diatoms with genera *Halamphora* and *Haslea* in the sample at 4.68 ka BP.

### Response of phytoplankton to temperature and zooplankton grazers

**Figure 4:**
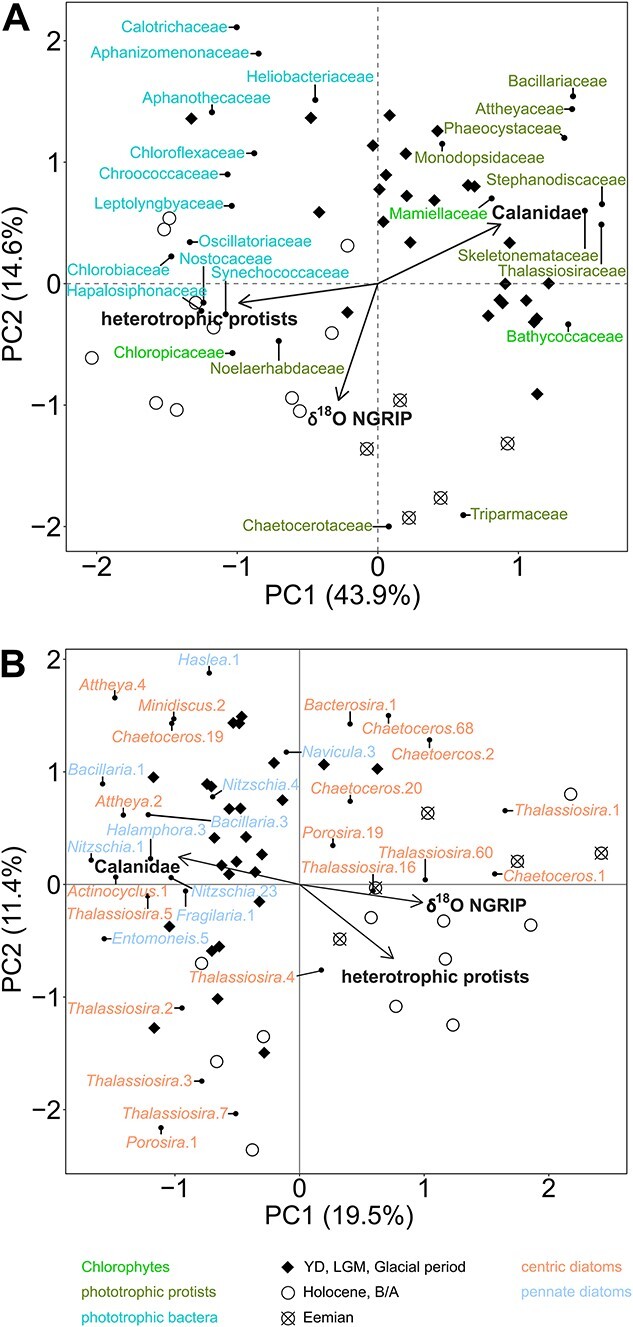
PCA of the phytoplankton community with environmental variables projected as vectors; (A) phytoplankton community on family level inferred from the shotgun approach; (B) diatom community on ASV level inferred from amplicon sequencing of the rbcL gene of diatoms; ASVs of the same genus are numbered arbitrarily; color of the taxa refers to their broader taxonomic unit (chlorophytes, prototrophic protists, and phototrophic bacteria in shotgun metagenomics; centric and pennate diatoms in diatom amplicon sequencing); samples are coded by symbol according to whether they are from a warm period—Holocene and B/A or Eemian—or from a cold period including the YD, the LGM, and the remaining last glacial period; the δ^18^O NGRIP climate record is published elsewhere [48]; *Calanidae* = Hellinger transformed relative abundances of *Calanidae* relative to all detected zooplankton; heterotrophic protists = sum of Hellinger transformed relative abundances of the heterotrophic protist families *Apusomonadidae* and *Salpingoecidae*.

The PCA of the shotgun sequencing phytoplankton data indicates a separation along the first axis (*x*-axis), dividing pico- and nano- from microsized phytoplankton (Fig. 4A). The second axis (*y*-axis) separates samples mainly along climatic conditions (δ^18^O NGRIP), clustering samples into warm and cool periods. The Eemian samples concentrate in the lower right quarter suggesting that the photoautotrophic community composition is intermediate between Holocene and glacial samples. The RDA of the zooplankton families, especially the heterotrophic protists, supports the clustering according to phytoplanktonic cell size along the first axis, whereas δ^18^O NGRIP, as a climate proxy, is mostly associated with community changes along the second axis. In total, 44.8% of the variance in the phytoplankton community composition can be significantly explained by the zooplankton community and the Northern hemisphere temperature variability (*P* = .001). The unique effect of heterotrophic zooplankton (families *Apusomonodidae* and *Salpingoecidae*) significantly explains 22.8% (*P* = .001) of the variance, whereas the relative abundance of *Calanidae* only explains 1.3% (*P* = .003). The heterotrophic zooplankton is associated with Holocene samples, which are dominated by picosized bacterioplankton. Northern hemisphere climate significantly explains 11.1% (*P* = .001) of the phytoplankton community variance.

In the diatom amplicon sequencing dataset, the first axis mainly separates warm (primarily ASVs of *Chaetoceros*, *Porosira*, and *Thalassiosira*) from cold period samples (primarily ASVs of *Attheya*, *Nitzschia*, and *Bacillaria*), supported by the results of the RDA which projects the δ^18^O NGRIP mainly along the first axis (Fig. 4B). Northern hemisphere climate and zooplankton together significantly explain 17.3% (*P* = .001) of the variance in the diatom community. The unique effect of δ^18^O NGRIP is 9.4% (*P* = .001) and of the heterotrophic zooplankton is about 9.4% (*P* = .001). No unique effect of *Calanidae* was computed.

## Discussion

### A shift from microphytoplankton to picosized bacterioplankton in the Holocene

**Figure 5:**
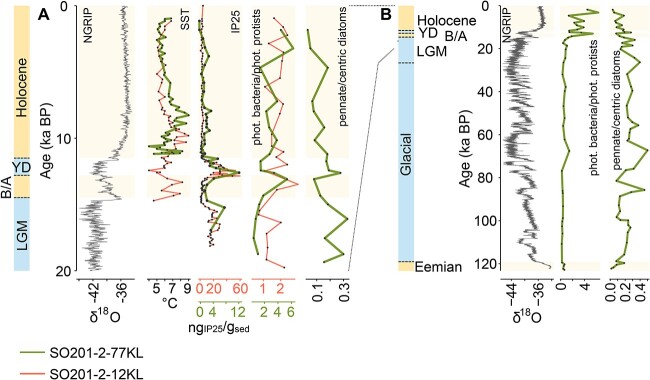
Environmental and community data from sediment core SO201-2-77KL (this study) and another Bering Sea sediment core SO201-2-12KL over the past 20 kyrs (A) and 124 kyrs (B); the δ^18^O NGRIP climate record is published elsewhere [48]; sea surface temperature (SST) reconstructions [30] calibrated in previous work [76]; the biomarker IP_25_ is used as a proxy for the presence of sea ice [31]; ratio of phototrophic bacteria:phototrophic protists of sediment core SO201-2-12KL [19]; warm periods (Holocene, B/A, Eemian) and cool periods (YD, LGM, and the last glacial period) are indicated on the left panel.

The reconstruction of the phytoplankton community composition in the western Bering Sea generally reveals a shift from microphytoplankton toward picosized bacterioplankton in the Holocene, including the B/A period (Fig. 2). These changes in the size structure of the phytoplankton community are likely to be explained by an increase of SST during warm periods (Fig. 5). Increasing SST resulted in sea ice decline (IP_25_ record in Fig. 5) [31, 33], subsequent freshening of the sea water, and enhanced thermohaline stratification [32]. Thereby, the oligotrophy of the surface layers during interglacial periods was increased [49], causing lower nutrient concentrations in spring and summer stratified water. Smaller picophytoplankton are favored over microphytoplankton due to a higher surface-to-volume ratio, which results in more effective nutrient utilization [50]. A comparative *sed*aDNA metagenomic study from an adjacent sediment core focusing on community changes during the last deglaciation (SO202–2-12KL, Fig. 1) supports our results ([19]; ratio phototrophic bacteria:phototrophic protists in Fig. 5). Furthermore, our findings of a shift in size structure over geological time are corroborated by short decadal time series [51], field observations from different ocean regions [5, 7], and from modeling studies [52], which suggest that it is not just a local phenomenon of the most recent past, but a long-term and potentially global trend.

During the last glaciation, sea ice extended further south [31, 33] providing suitable environmental conditions for sea ice-associated phytoplankton in the western Bering Sea. Both *sed*aDNA approaches indicate a glacial phototrophic community dominated by microphytoplankton within the diatom families *Attheyaceae*, *Bacillariaceae*, *Thalassiosiraceae*, and putatively cold-adapted *Chaetoceros* lineages. However, *Chaetoceros* is generally more abundant during the warm interglacials, although some cold-adapted lineages were identified at the ASV level. The presence of different ASVs of *Chaetoceros* in different thermal regimes suggests that species or populations within the genus *Chaetoceros* are able to successfully adapt to changing environmental conditions [25]. Also, our data indicate the occurrence of picosized *Bathycoccaceae* and *Mamiellaceae*, as well as nanosized *Phaeocystaceae* during the glacial period, along with an increase in sea ice-associated pennate diatoms (ratio pennate:centric diatoms in Fig. 5). *Bathycoccaceae* and *Mamiellaceae* include cold-adapted algae (e.g. *Bathycoccus*) able to survive under prolonged dark winter conditions in the Arctic Ocean [53-55] and *Phaeocystaceae* blooms are associated with seasonal sea ice in the modern Arctic Ocean [56].

A glacial-like community is also detected in one exceptional sample in the Holocene (4.68 ka BP) and might be a result of decreasing temperatures during the late Holocene. There is evidence from sea ice reconstructions in the Bering Sea [30] and temperature reconstructions from the Fram Strait [57] that cold conditions were prevalent during ~5–3 ka BP.

### Eemian phytoplankton community dominated by picosized eukaryotes

Both *sed*aDNA approaches in this study indicate differences in the phytoplankton composition of the Holocene and the Eemian. The most intensive warming period during the Eemian (122–128 ka BP; [36]) is covered here by three samples. The shotgun community composition in these samples indicates highest relative abundance of *Triparmaceae* (mainly *Triparma laevis*; Supplementary Data 3) and a strong reduction of sea ice-associated families such as *Attheyaceae*, *Bacillariaceae*, and *Phaeocystaceae* compared to the glacial period. Furthermore, sea ice diatoms such as *Attheya* and *Haslea* completely disappear from the amplicon sequencing data. *Triparma laevis* nowadays occurs in the western North Pacific at lower latitudes (45–35°N) and optimally grows in surface waters between 5 and 10°C, thus adding an important component of the phytoplankton community in the temperate Pacific [58]. High abundance of this taxon during the Eemian provides evidence of temperate conditions in the western Bering Sea during that time. However, a strong increase in nonsilicified bacterioplankton, as seen in the Holocene, is not detected.

Samples from the late Eemian show partly an intermediate state between glacial and Holocene samples (Fig. 4A). A high relative abundance of sea ice-associated diatoms such as *Attheya* and *Haslea* in the amplicon sequencing dataset as well as high abundances of *Attheyaceae* and the cold-adapted chlorophyte family *Bathycoccaceae* in the shotgun dataset suggest cooler conditions during the late Eemian. A modeling study supports the occurrence of cooling events during the Eemian, e.g. the late Eemian Värriö event [59], that might have caused plankton shifts toward cold-adapted lineages thereby disrupting the expected signal of warm-adapted communities. However, the absence of phototrophic bacteria, which is a major component of the Holocene phytoplankton, limits an analogy to communities of primary producers in the Bering Sea under enhanced future warming and leads to the rejection of our hypothesis that interglacials are generally characterized by a community change toward photoautotrophic picosized bacteria.

### Reduction of crustaceous zooplankton and increase of heterotrophic protists in warm periods

The *sed*aDNA shotgun approach identifies major taxonomic components of the zooplankton community and suggests a decrease in crustaceous zooplankton within the families *Calanidae* and *Metridinidae* (copepods) during warm periods (Fig. 2B). Copepods are a dominant zooplankton group in northern and polar oceans and show increased abundance with the occurrence of microphytoplankton, whereas their abundance declines with an increase in temperature, salinity, and abundance of picophytoplankton [60]. *Metridinidae*, a dominant family detected in our sedimentary record, is also present in the modern western Bering Sea where their abundance is directly linked to diatom blooms. With extended feeding on microzooplankton, they are able to survive the winter season [61]. Zooplankton links phytoplankton primary productivity with energy transfer to higher trophic levels [17]. Our *sed*aDNA data show a response of the zooplankton community composition along with changes in the phytoplankton community (Fig. 2). For example, copepods might feed on low-quality food due to the shift from micro- to picosized phytoplankton during warm periods. Consequently, body size decreases and egg production can be reduced [16, 62], resulting in the reduction of copepod abundance with increasing bacterioplankton (Figs 2 and 5). Also, increased temperature can affect the copepod community with a decline of nauplius abundance and a shift toward a juvenile dominated community structure under enhanced temperature conditions [63]. The exceptional Holocene sample at 4.68 ka BP, showing high relative abundances of sea ice-associated algae, also shows higher numbers of copepods compared to other Holocene samples.

Generally, the proportion of heterotrophic protists increases in our record in balance with the decline of copepod abundance during warm interglacials. In contrast to (sub)arctic copepods, heterotrophic protists display a broad thermal tolerance [64] and feed on bacteria [65, 66], among other organisms. We detected higher picosized bacterioplankton abundances, particularly during the Holocene, and picosized *Triparmaceae* proportions during the Eemian than during the glacial periods. Potentially, such an increase in the availability of picosized algae provided better conditions for heterotrophic protists and enhanced their proliferation during warm periods.

### Consequences of plankton community shifts for the food web and carbon export through time

Abiotic environmental changes, being combined effects of changes in light availability due to changes in sea ice cover, temperature, the elemental composition of the seawater, and the concentration of dissolved CO_2_, are known to regulate the ecological community structure, and hence primary productivity [7, 11]. Our study finds that primary productivity in the western Bering Sea under glacial conditions is dominated by sea ice-associated diatoms, *Phaeocystaceae*, and cold-adapted eukaryotic chlorophytes. Pennate diatoms and haptophytes are known to form seasonal algae blooms and efficiently fix CO_2_ below the sea ice and under limited light conditions in the modern Arctic Ocean [56, 67], which we assume to resemble the glacial community composition in the Bering Sea. The strong trophic link between phototrophic protists and copepods results in the coupling of abundance trends in modern marine ecosystems [68] as well as in our *sed*aDNA record. The aggregation of algae and the grazing by copepods facilitates carbon export to deeper water depth due to the production of fast-sinking fecal pellets [49, 69, 70]. However, this effective mechanism of CO_2_ export during cold periods occurs in a generally low-productivity environment [32] due to cold SST, reduced light availability caused by enhanced sea ice coverage, and lower atmospheric pCO_2_ levels.

With the onset of the Holocene, our study shows a change from glacial communities to the dominance of bacterial picophytoplankton, mainly cyanobacteria, in the western Bering Sea until our youngest sample 1.82 ka BP putatively triggered by climate warming. This shift in the size structure of the phytoplankton community coincides with an increase in productivity compared to the glacial period that was reconstructed elsewhere [32]. However, pico- and nanophytoplankton reveal slower sinking velocities relative to larger phytoplankton cells, and the Holocene bacterioplankton also lack ballast provided by inorganic cell walls [71], thereby fueling the microbial loop by a rapid turnover. Consequently, the full potential of possible carbon export to the deep sea might not be realized in this period. Moreover, a dominance of bacterioplankton is shifting the community of grazers, which is supported in our data by an increased abundance of bacterivorous (heterotrophic) protists during the warm periods. In contrast to grazing by copepods, grazing by heterotrophic protists facilitates organic matter degradation [72] and carbon recycling in the water column, thereby weakening the benthic-pelagic coupling and carbon export rates [17, 73]. Our data support a decline in the abundance of large diatoms and copepods (mainly *Calanidae*) during generally warmer periods (Eemian, B/A, and Holocene) and an increase in the relative abundance of heterotrophic protists, putatively contributing to reduced carbon export. These findings are in accordance with recent evidence that under sea ice-free conditions, the sinking rates of particulate organic carbon are also reduced in the modern Bering Sea [74].

Although the Holocene and the Eemian periods both show increased relative abundances of nano- and picosized phytoplankton taxa, the carbon export in the Eemian was putatively higher than in the Holocene due to a different composition of a smaller-sized community. Although Holocene pico- and nanophytoplankton is represented mainly by bacterioplankton, the Eemian nano- and picosized community is represented by silicifying *Triparmaceae*. The additional ballast provided by the inorganic cell walls of *Triparmaceae* possibly resulted in faster sinking rates and higher carbon export [71]. Moreover, diatoms are more abundant in the Eemian than in the Holocene and may contribute to enhanced carbon export rates.

However, quantifying the Bering Sea’s potential as a carbon sink using our *sed*aDNA record is not possible, as it only provides information about the community composition. For a quantitative assessment, more abiotic (e.g. atmospheric pCO_2_ levels, water temperature, oxygenation state of the water column) and biotic variables (e.g. total productivity, trophic interactions, microbial activity) would have to be studied in more detail.

In summary, high-latitude marine ecosystems are particularly affected by global climate change. Increasing ocean temperatures, expression of seasonality, nutrient supply and utilization, and the retreat of sea ice mainly affects the timing of primary productivity and the trophic coupling. The related community changes toward a plankton community dominated by picosized phytoplankton and its consumers in subarctic and Arctic ocean areas will have consequences for higher trophic levels, thereby altering ecosystem function and stability. For reliable predictions of the future Bering Sea’s community composition and its implications for the carbon cycle, possible synergistic and antagonistic effects have to be studied in more detail. Although much research has been done on recent ocean warming and its effects on the plankton community, our study reveals that the changes in the community structure observed in the modern ocean appear to magnify a long-term trend of decreasing phytoplankton size in the current interglacial. In this extent, this seems not to be a common feature of interglacials (e.g. Holocene vs. Eemian), which could be explained by different temperature trajectories during these warm phases.

## Supplementary Material

BeringSea_Supplementary_information_revised3_2023_12_14_wrad006

## Data Availability

Coring location and chronostratigraphy are available [31, 32]. Raw DNA shotgun data (Bioproject number PRJE866300) and raw DNA amplicon sequencing data (Bioproject number PRJEB66201) have been deposited in the European Nucleotide Archive (ENA) using the data brokerage service of the German Federation for Biological Data (GFBio) [77], in compliance with the Minimal Information about any (X) Sequence (MIxS) standard [78]. Input data, bioinformatic scripts, and R scripts are available under https://doi.org/10.5281/zenodo.10064386. Final datasets are also provided as Supplementary files.
