# Correction to: Plankton community changes during the last 124 000 years in the subarctic Bering Sea derived from sedimentary ancient DNA

**DOI:** 10.1093/ismejo/wraf241

**Published:** 2025-11-06

This is a **correction** to:

Stella Z Buchwald, Ulrike Herzschuh, Dirk Nürnberg, Lars Harms, Kathleen R Stoof-Leichsenring, Plankton community changes during the last 124 000 years in the subarctic Bering Sea derived from sedimentary ancient DNA, *The ISME Journal*, Volume 18, Issue 1, January 2024, wrad006, https://doi.org/10.1093/ismejo/wrad006

In the originally published version of this manuscript, an incorrect ENA repository number was listed within the Data Availability section - The raw DNA shotgun data (Bioproject number PRJE866300). The correct number of the Bioproject is PRJEB46821.

These details have been corrected only in this **correction notice** to preserve the published version of record.

