# Supplementary material for: Plankton community changes during the last 124 000 years in the subarctic Bering Sea derived from sedimentary ancient DNA
Source: ISME J. 2024 Jan 10;18(1):wrad006. doi: 10.1093/ismejo/wrad006 (PMC10811732; doi:10.1093/ismejo/wrad006)
Supplement: BeringSea_Supplementary_information_revised3_2023_12_14_wrad006 [file beringsea_supplementary_information_revised3_2023_12_14_wrad006.pdf]

## Supplementary Information

Plankton community changes during the last 124 000 years in the subarctic Bering Sea derived from sedimentary ancient DNA

**Authors**

Stella Z. Buchwald[1†], Ulrike Herzschuh[1,2,3], Dirk Nürnberg[4], Lars Harms[5], Kathleen R. Stoof-Leichsenring[1*]

**Affiliations**

[1]Alfred Wegener Institute Helmholtz Centre for Polar and Marine Research, Polar Terrestrial Environmental Systems, D-14473 Potsdam, Germany

[†]current address: Universität Hamburg, Institute for Geology, D-20146 Hamburg, Germany

[2]University of Potsdam, Institute of Biochemistry and Biology, D-14476 Potsdam, Germany

[3]University of Potsdam, Institute of Environmental Sciences and Geography, D-14476 Potsdam, Germany

[4]GEOMAR Helmholtz Centre for Ocean Research Kiel, Ocean circulation and climate dynamics, D-24148 Kiel, Germany

[5]Alfred Wegener Institute Helmholtz Centre for Polar and Marine Research, Data Science Support, D-27568 Bremerhaven, Germany

*corresponding author (Kathleen.Stoof-Leichsenring@awi.de)

## Expanded Material and Methods

**Sample material**

During the R/V *Sonne* cruise SO201-KALMAR Leg 2 in 2009, the sediment core SO201-2-77KL (77KL) was recovered with a piston corer from the Shirshov Ridge (56.3305°N, 170.6997°E) in a water depth of 2 135 m (1). It has a total length of 11.78 m. Thesedimentation rate of 3-30 cm/kyr on average allows for a centennial temporal resolution. After retrieval, the core was cut in 1 m pieces, packed with plastic foil in core boxes to prevent drying and reduce the risk of contamination of the sediments with modern DNA or other sediment samples.  Cores were stored cool (4°C) and dark in a dedicated core storage room at GEOMAR Helmholtz Centre for Ocean Research in Kiel.

**Extended description of the age-depth model**

The chronostratigraphical approach for sediment core SO201-2-77KL includes accelerator mass spectrometry (AMS) radiocarbon ($^{14}C$) dating of planktonic foraminifera, benthic $\delta^{18}O$ stratigraphy, magnetostratigraphy, and the tuning of high-resolution core logging data (color b*, XRF scanning) to various climate records. The stratigraphic framework for the last 20 kyr mainly based on AMS$^{14}$C dating, is provided elsewhere (2) and age control back to 120 ka BP was added (1).  A detailed description of the chronostratigraphy is presented in the Supplement Information and in the original publications (1, 2).

AMS$^{14}$C-ages were determined on the planktonic foraminiferal species *Neogloboquadrina pachyderma* sinistral (*N. pachyderma* sin.) from the 125–250 µm size fraction (Table S1). The AMS$^{14}$C-ages were converted into calibrated 1-sigma calendar ages using the calibration software Calib Rev 6.0 (3) with the Intcal09 atmospheric calibration curve (4), taking into account a constant reservoir age.

Table S1. AMS$^{14}$C-ages of sediment core SO201-2-77KL with calibrated calendar age $\pm$ 1-sigma (years) and a reservoir age correction of 700 years. The datings were provided by the

National Ocean Science Accelerator Mass Spectrometry Facility (NOSAMS, Woods Hole, USA) and at the Leibniz-Laboratory for Radiometric Dating and Isotope Research at Kiel University (Germany).

| Laboratory Number | Sediment Core | Core Depth (cm) | Radiocarbon Age (years) | Calendar Age ±1σ (years) | Reservoir Age (years) |
|---|---|---|---|---|---|
| OS-85671 | SO201-2-77KL | 105 | 9570 ± 45[b] | 10051–10152 | 700 |
| OS-85658 | | 115 | 10450 ± 40[c] | 11174–11222 | 700 |
| OS-90700 | | 155 | 11500 ± 50 | 12608–12727 | 700 |
| OS-85657 | | 167–170 | 12750 ± 50 | 13823–13967 | 700 |
| OS-85664 | | 180 | 13200 ± 45 | 14501–14945 | 700 |

The age control beyond the radiocarbon datings is primarily based on the graphic correlation between the color b* record of core SO201-2-77KL to those of adjacent core SO201-2-85KL, which is slightly north of core SO201-2-77KL (1). The chronostratigraphy of core SO201-2-85KL is based on a tight correlation of the color b* record to the Sanbao and Hulu stalagmite $\delta^{18}O$ records (5, 6) and the Greenland climate $\delta^{18}O$ reference record (NGRIP members, 2004; GICC05 timescale; 7). In addition, the core SO201-2-85KL relative paleointensity (RPI) record is tuned to the PISO-1500 paleomagnetic reference record (8), by considering the Laschamp (~42 ka BP), Norwegian–Greenland Sea (~65 ka BP), and Blake (~117 ka BP) paleomagnetic excursions. Further age control was derived from the comparison of benthic $\delta^{18}O$ values with the global reference stack LR04 (9). The age models are supported by spectral analysis (AnalySeries 2.0; 10) of the color b* and benthic $\delta^{18}O$ records. Dominant cyclicities of ~23 and ~39 kyrs are present, which match frequencies of orbital precession (0.047 ± 0.005 $kyr^{-1}$) and obliquity (0.025 ± 0.0015 $kyr^{-1}$) within appropriate bandwidths.

Therefore, as 1-2 cm of sediment were used per sample, the respective communities are time-averaged. Due to variable sedimentation rates between ~3-30 cm/kyr with lower rates during the glacial period than during interglacial periods (1), Holocene samples represent ~50-100 years, glacial samples represent ~350 years and Eemian samples represent ~100 years.

**Core sampling and DNA extraction**

The core SO201-2-77KL was sampled at 54 sections over its full length in the sediment lab facilities of the GEOMAR Helmholtz Centre for Ocean Research Kiel without any molecular laboratories in the same building. Subsampling was performed wearing a full body sterile lab coat and sterile instruments for cutting the sediments. Surfaces and instruments were cleaned with DNA ExitusPlus (Applichem) and technical Ethanol. The outer surface of the sediment core was removed and samples were taken only from the inner and untouched part of the sediment. Samples were stored in sterile tubes and shipped under cool

conditions to the Alfred Wegener Institute (AWI), Potsdam (Germany), for further processing in dedicated paleogenetic DNA laboratories. Ensuring that samples are free of modern DNA contamination or cross-contamination while subsampling under non-sterile conditions at GEOMAR, samples were again subsampled under clean-room conditions with similar equipment as described above. Therefore, the outer surfaces of the subsamples were removed with sterile knives and tweezers. Several cuts with sterile instruments were done to remove each side of the sediment sample. All working steps were performed under a dedicated subsampling UV hood, which is a small extra working bench placed in the paleogenetic DNA laboratories that use UV light to decontaminate the working surface. The DNA extraction was performed under a dedicated extraction UV hood. The DNA extraction was done using the DNeasy PowerMax Soil Kit (Qiagen, Germany) with 8-10 g of sediment per sample. Nine sediment samples and an additional extraction blank were extracted per batch following the manufacturer's recommendations with few modifications: adding 400 µL proteinase K (20 mg/ $mL^{-1}$) and 100 µL dithiothreitol (DTT, 5M) to C1 solution, vortexing on highest speed (~2,800 rpm) for 10 min and incubating samples overnight in a rotating system at 56°C. DNA extracts were concentrated and purified with the GeneJET PCR Purification KIT (ThermoFisher Scientific). DNA concentrations were measured with the Qubit dsDNA BR Assay Kit (Invitrogen) on a Qubit 4.0 fluorometer (Invitrogen). The DNA concentration of all extraction blanks was below detection limit (< 0.1 mg/ $\mu L^{-1}$). Extraction blanks were not concentrated with GeneJET PCR Purification KIT (ThermoFisher Scientific).

**Indexing PCR for the metagenomic shotgun approach**

Libraries were quantified via quantitative PCR (qPCR) to estimate the number of cycles for library indexing amplification. For indexing PCR, a master mix was prepared containing 57 µL VE water, 10 µL AccuPrime Pfx reaction mix (x10; Life Technologies) and 1 µL AccuPrime Pfx Polymerase (2.5 U/µL) per sample; 24 µL of library sample were added to the master mix. A unique combination of P5 and P7 primers was added to each sample and each primer was only used once; 4 µL of each primer were added per sample. Depending on the results of the qPCRs, the indexing PCRs ran between 10 and 13 cycles. Extraction blanks and library blanks ran with the lowest number of cycles calculated for the respective batch, ranging between 10 and 12 cycles. The fragment length of the libraries was measured with TapeStation4200 (Agilent Technologies, California).

**PCR with rbcL primer for the diatom amplicon-sequencing approach**

For diatom amplicon-sequencing, all 54 samples were PCR amplified by targeting a short and specific fragment of the gene of the large subunit of the ribulose-1,5-bisphosphate

carboxylase/oxygenase (rbcL) in diatoms (11). Both forward and reverse primers were designed with a unique sequence of 8 bp on the 5'-end and a random NNN suffix was added. For demultiplexing of the sequencing data, each sample, extraction blank and PCR non-template control was tagged with a unique primer combination. A master mix containing 12.75 µL VE water, 2.5 µL 10X HiFi Taq DNA Ligase Buffer, 2.5 µL dNTPs (2.5 mM; Invitrogen), 1 µL Bovine Serum Albumin (BSA; 20 mg/mL), 1 µL $MgSO_4$ (50 mM) and 0.25 µL Platinum Taq High Fidelity DNA Polymerase (Invitrogen) per sample was produced and 1 µL tagged forward primer *rbcL_705F* (5' NNN-8 bp tag-AACAGGTGAAGTTAAAGGTTCATAYTT 3'), 1 µL tagged reverse primer *rbcL_808R* (5' NNN-8 bp tag-TGTAACCCATAACTAAATCGATCAT 3') and 3 µL template DNA were added. The PCR reaction was conducted in a Biometra ThermoCycler (Jena Analytik) with the setting: 5 min at 94°C (preheating), 50 cycles of 30 s at 94°C (denaturation), 30 s at 49°C (annealing) and 30 s at 68°C (elongation), followed by 10 min at 72°C (final extension). PCR success was checked by gel electrophoresis on a 2% agarose gel and repeated until three replicates showed DNA bands at the expected position in the gel.

**Damage pattern analysis**

Damage pattern analyses for selected phytoplanktonic (*Synechococcus*, *Fragilariopsis cylindrus*, *Bathycoccus prasinos*) and zooplanktonic (*Eurytemora affinis*, *Salpingoeca rosetta*, *Thecamonas trahens*) taxa were performed by the automated HOPS 0.34 pipeline (12) on only merged read data against the non-redundant nucleotide database (built for malt alignment used by HOPS). Results for damage pattern analyses are given for default and ancient classified reads of selected taxa showing the proportion of C to T substitutions compared to the other substitutions (noise) in the first ten positions of the affected reads against a modern genomic reference. Results are presented below in Figs. S8-S9.

**Bioinformatic analysis with OBITools**

The raw paired-end sequencing data of the diatom amplicon-sequencing approach (202 PCR products, including three replicates per sample, 18 replicates of extraction blanks and 22 PCR non-template controls) was analysed with the Python package OBITools 3.0.1 (13). Paired-end reads were merged with the function *obi alignpairedend*, filtered for the respective primer combination with *obi ngsfilter*, grouped *obi uniq* and taxonomically classified with *obi ecotag* and *obi annotate* based on their similarity to the rbcL-EMBL nucleotide reference database. This database was conducted with *ecoPCR* (5) using the non-redundant nucleotide database, released in April 2020, for an *in silico* PCR approach that allowed 5 mismatches between primer and target sequences.

**Filtering the diatom amplicon-sequencing dataset**

The dataset that results from the OBITools pipeline was further filtered stepwise on the level of Amplicon Sequence Variants (ASVs). During the filtering, only ASVs were kept that fulfill the following criteria: The taxonomic assignment is based on 96-100% similarity to an entry in the EMBL reference database and at minimum up to the division of Bacillariophyta (diatoms). The ASV has a minimum read count of 100 summarized over all replicates and a minimum read count of 10 within a replicate. The ASV is present in at least three out of all replicates. If the ASV in a replicate has no counts labeled with *head*, the *internal* counts make up less than 50% of all counts within this replicate. After filtering, the counts of the three replicates of a sample were aggregated.

**Expanded statistics**

For the RDA, stable oxygen isotope data from the North Greenland Ice Core Project ($\delta^{18}O$ NGRIP; 7) were used as a proxy for northern hemisphere climate. The effect of the northern hemisphere climate on the explained variance within the total phytoplankton community (shotgun approach) or the diatom community (diatom amplicon-sequencing) was investigated. Where no data were available at the same age as the sample age, values were interpolation linearly from the nearest neighbors (14).

Because not all samples of the diatom amplicon-sequencing approach have also been processed in the metagenomic shotgun approach, the PCA and RDA of the metabarcoding dataset run only on those samples for which zooplankton abundance data were available from the shotgun approach. The explanatory variables were projected as vectors in the graphical illustration of the PCA using the function *envfit*. The *p* values of explained variance in the phytoplankton community composition were calculated by performing an ANOVA with the function *anova* on the output of RDAs with a single explanatory environmental variable (for unique effect) or all explanatory variables (combined effect of all variables) restricting the community composition.

For the assessment of DNA quality in our samples we performed spearman correlation analysis between wet lab data (DNA concentration after extraction and GeneJET purification, DNA library concentration, library average fragment length) and bioinformatic data (raw and filtered read counts) against sample age and environmental proxies including NGRIP, TC, TOC, TN, $CaCO_3$, and color b* (Supplementary data 5). Environmental proxies were interpolated linearly to retrieve data points for all required sample ages. All correlation analyses were performed in R (version 4.1.2) using the *Hmisc* package (version 4.7-2) and

*rcorr* and *corrplot* function (sig.level = 0.05). Multiple line plots were produced with ggplot2 (version 3.4.0).

## Expanded Results

### *seda*DNA quality assessment

The quality of the shotgun *seda*DNA data was estimated by comparing wet lab results: sample weight (in g), DNA concentrations (DNA concentration of the extraction in ng/µL, DNA concentration of the extract after GeneJET purification in ng/µL, DNA concentration of the DNA library after ssDNA library preparation for shotgun in ng/µL) and fragment length of the DNA libraries (in bp); bioinformatic results including DNA read counts (results of the bioinformatic analyses including number of raw reads and taxonomically classified reads) against sample age and additional environmental proxies ($\delta^{18}O$ NGRIP – proxy for reconstructed Northern hemisphere climate; TC (total carbon in wt %), TOC (total organic carbon in wt %), TN (total nitrogen wt %), $CaCO_3$ (in wt %), color b* (proxy for biogenic silica) from the KL77 sediment core (Supplementary data 5).
The variation in DNA concentration estimates (Figure S1), given a relatively constant input weight of wet sediment (median = 8.3 g per extraction), is negatively correlated with the sample age (Figure S2, Supplementary data 5). In contrast, there is no significant correlation between the DNA library concentration, while we detected a weak negative relationship between the library fragment length and the sample age. The geochemical proxies measured in the sediment core (Figure S3), including total carbon (TC), total nitrogen (TN), $CaCO_3$ and color b* decrease with sample age and are therefore negatively correlated with sample age (Figure S2) and at the same time slightly positively correlated with DNA concentration.
The variations in the number of reads (raw or taxonomically classified read counts) derived from shotgun sequencing (Figure S4) is not correlated with sample age (Figure S2).
There is a slight positive correlation between the raw read numbers and total nitrogen (TN) and also between the classified reads and total carbon (TC).
Conclusively, the concentration of DNA from sediments depends mainly on the sample age, whereas the quality (read counts derived from sequencing) does not depend on the age of the sample, but is rather positively correlated with the proportion of carbon and nitrogen in the sediments.

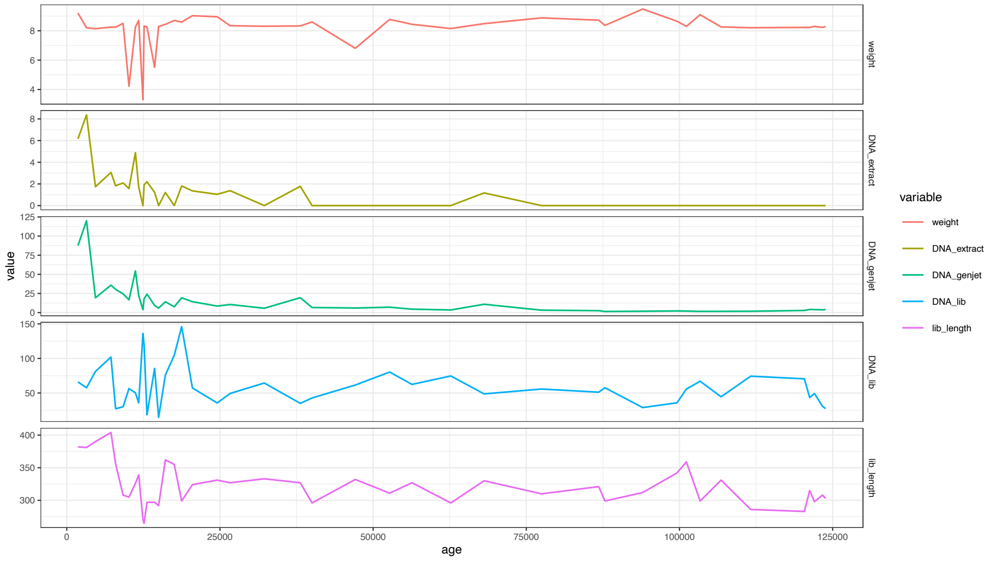


Figure S1: Variation in weight (sample weight in g), DNA_extract (DNA concentration of the extraction in ng/µL), DNA_genejet (DNA concentration of the extract after GeneJET purification in ng/µL), DNA_lib (DNA concentration of the DNA library after ssDNA library preparation for shotgun in ng/µL) and lib_length (fragment length of the DNA libraries including adapters in bp) across the 42 sample ages.

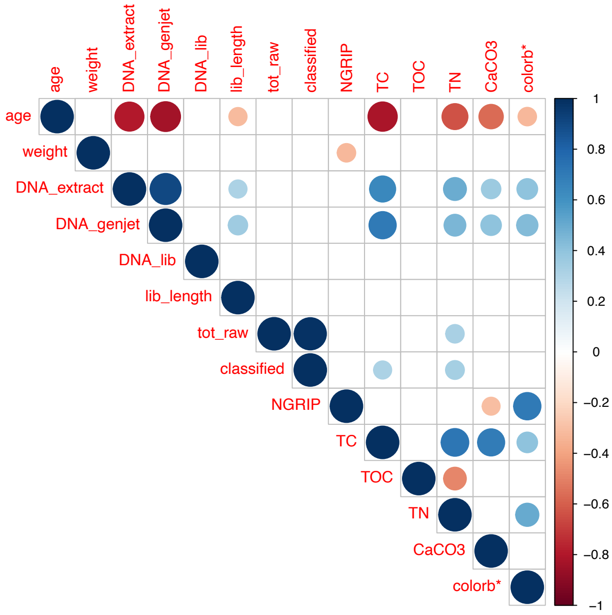


Figure S2: Correlation plot for the shotgun dataset (42 sediment core samples) with pairwise comparison between the following parameters: “age” (sample age in years), “weight” (sample weight in g), “DNA_extract” (DNA concentration of the extraction in ng/µL), “DNA_genejet” (DNA concentration of the extract after GeneJET purification ng/µL), “DNA_lib” (DNA concentration of the DNA library after ssDNA library preparation for shotgun ng/µL), lib_length (average fragment length of the DNA library extracted from the TapeStation4200 results in bp), “tot_raw” (raw number of read pairs), “classified” (filtered and classified reads), “NGRIP” ($\delta^{18}O$ climate proxy), “TC” (total carbon in wt %), “TOC” (total organic carbon in wt %), TN (Total nitrogen wt %), $CaCO_3$ (in wt %) and “color b*”. Values

used for the correlation analyses are given in the Supplementary data 5. The colored circles indicate a significance threshold of a $p$ value < 0.05, blue circle indicate positive, whereas red circles indicate negative correlations. The correlation coefficient is given in the right panel.

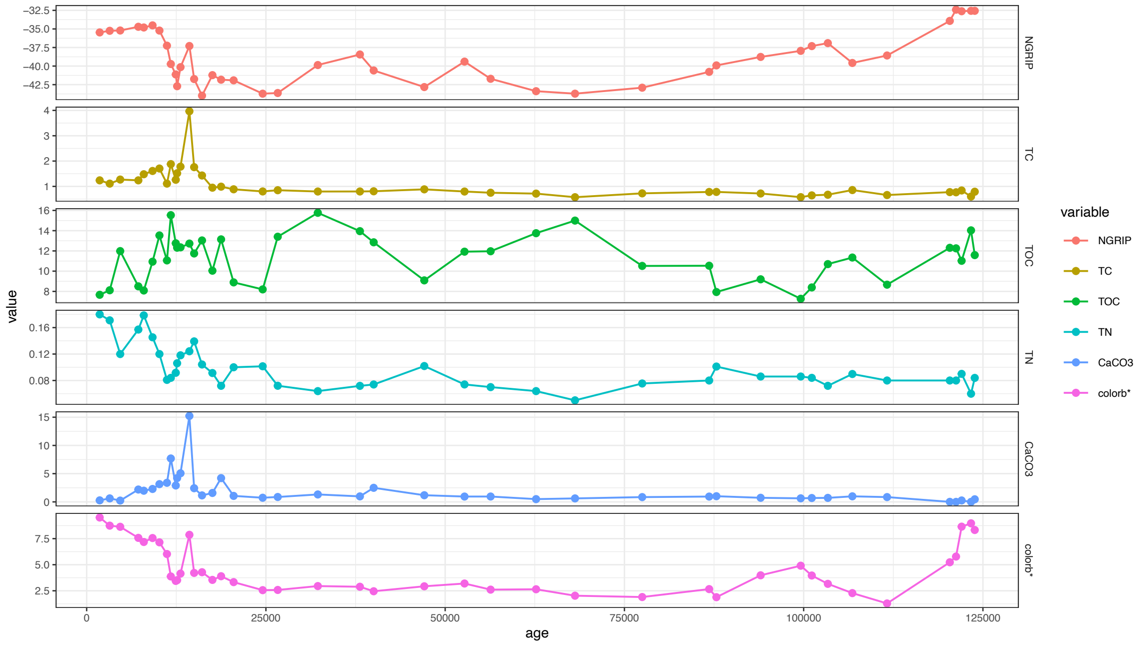


Figure S3: Variations in $\delta^{18}O$ NGRIP, total carbon (TC in wt %), total organic carbon (TOC in wt %), total nitrogen (TN), calcium carbonate $CaCO_3$ (in wt %) and color b* across the 42 sample ages.

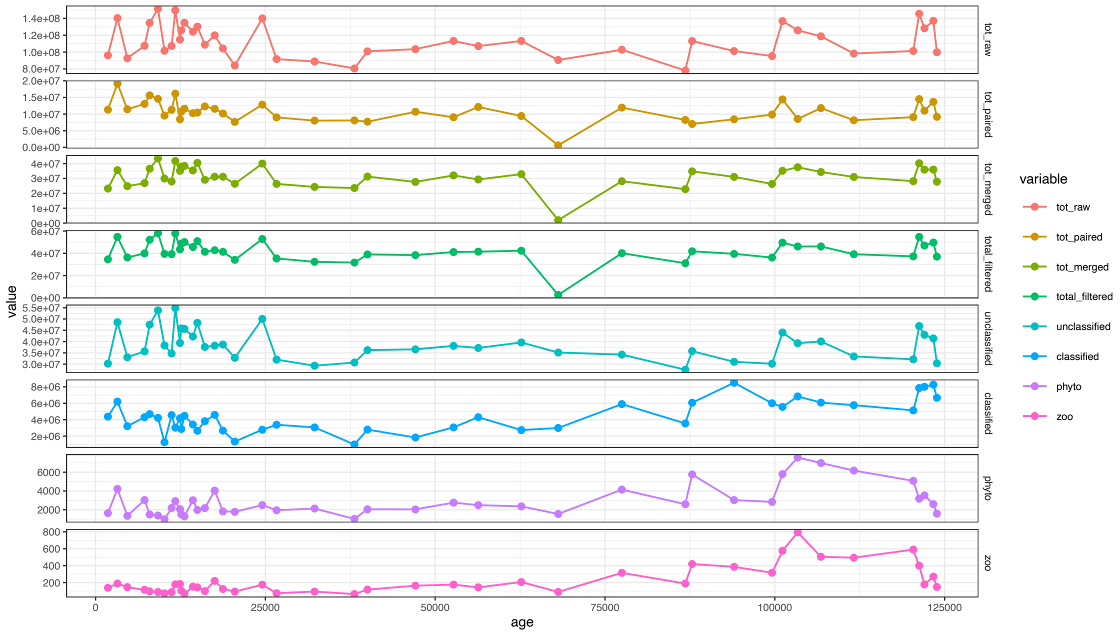


Figure S4: DNA sequence read numbers in read pairs across the 42 sample ages.

“tot_raw” (number of total raw reads pre trimming),”tot_paired” (number of total paired reads after trimming), "tot_merged" (number of total merged reads after trimming), "total_filtered" (number of total paired and merged reads after trimming), "unclassified" (number of unclassified reads after kraken classification nt0.2) "classified" (number of classified reads after kraken classification nt0.2), "phyto" (number of classified reads after phytoplankton family selection), "zoo” (number of classified reads after zooplankton family selection).

The quality of the amplicon-sequencing *seda*DNA (see read counts per sample before and after resampling in Supplementary data 6 & 7, Figure S5) was estimated by comparing wet lab results: sample weight (in g), DNA concentrations (DNA concentration of the extraction in ng/µL, DNA concentration of the extract after GeneJET purification in ng/µL; bioinformtatic results including raw count after amplicon-sequencing and read count after taxonomic filtering for diatoms (Figure S6) against sample age and additional environmental proxies ($\delta^{18}O$ NGRIP – proxy for reconstructed Northern hemisphere climate; TC (total carbon in wt %), TOC (total organic carbon in wt %), TN (total nitrogen wt %), $CaCO_3$ (in wt %), color b* (proxy for biogenic silica) (see Supplementary data 8).

DNA concentrations, like shown in the shotgun data set (DNA extracts are identical in shotgun and metabarcoding approach), indicate a negative correlation with sample age (Figure S7), like the diatom read number. Diatom read counts are negatively correlated with NGRIP, which indicates a lower diatom read count during periods of warmer climate. Positive correlations are identified between diatom read count and TC, TOC and $CaCO_3$.

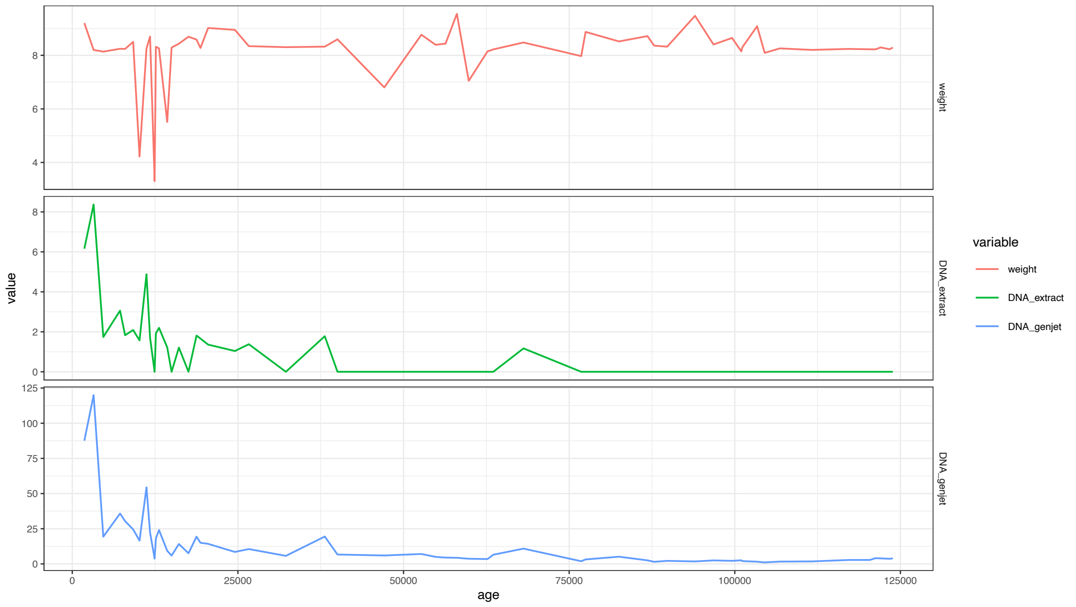


Figure S5: Variation in weight (sample weight in g), DNA_extract (DNA concentration of the extraction in ng/µL) and DNA_genejet (DNA concentration of the extract after GeneJET purification in ng/µL across the 54 sample ages.

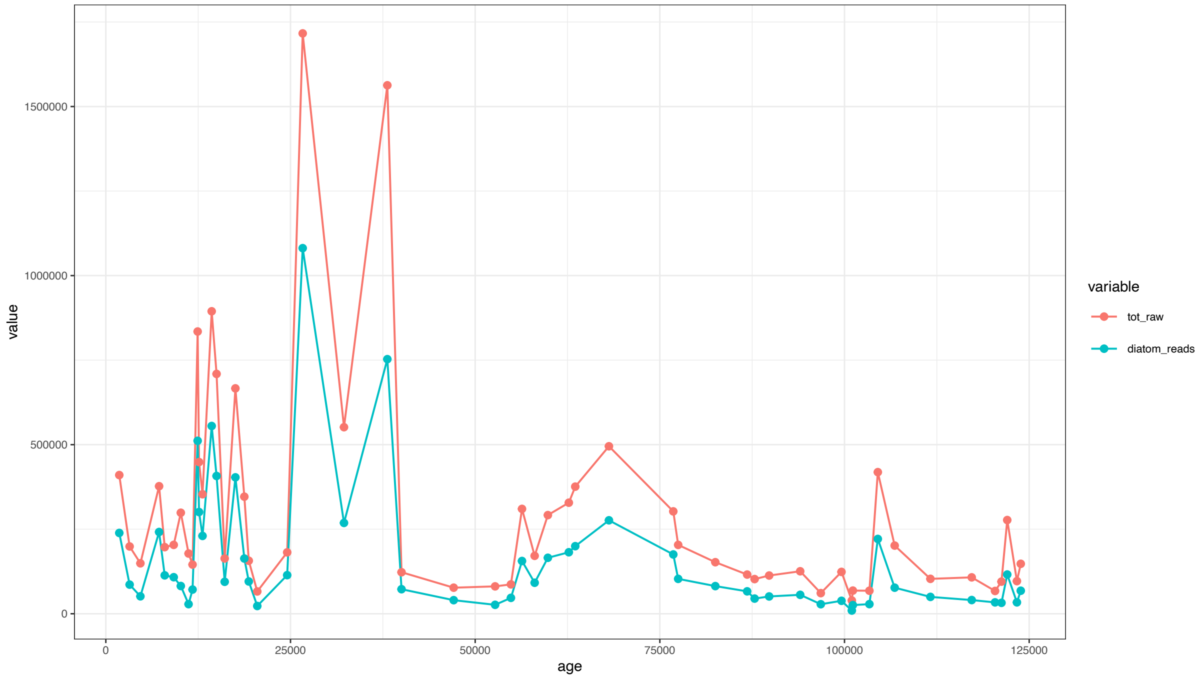


Figure S6: Sequence read numbers from the metabarcoding approach with “tot_raw” (total raw sequence reads merged for all three PCR replicates) and “diatom_reads” (total diatom read counts merged for all three PCR replicates) across the 54 sample ages.

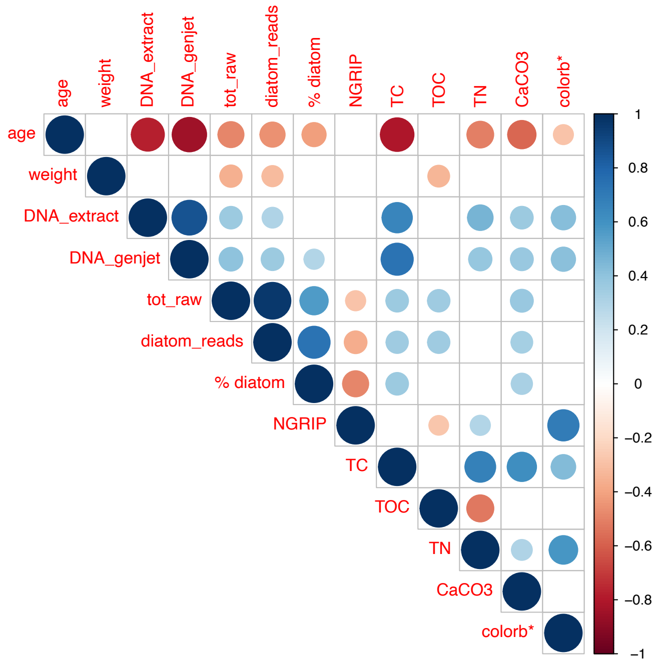


Figure S7: Correlation plot for the metabarcoding dataset (54 sediment core samples) with pairwise comparison between the following parameters: “age” (sample age in years),

“weight” (sample weight in g), “DNA_extract” (DNA concentration of the extraction in ng/µL), “DNA_genejet” (DNA concentration of the extract after GeneJET purification ng/µL), “tot_raw” (total raw sequence reads merged for all three PCR replicates) and “diatom_reads” (total diatom read counts merged for all three PCR replicates), “NGRIP” ($\delta^{18}O$ climate proxy), “TC” (total carbon in wt %), “TOC” (total organic carbon in wt %), TN (Total nitrogen wt %), $CaCO_3$ (in wt %) and “color b*”. Values used for the correlation analyses are given in the Supplementary data 8. The colored circles indicate a significance threshold of a *p* value < 0.05, blue circle indicate positive, whereas red circles indicate negative correlations. The correlation coefficient is given in the right panel.

## Damage pattern analysis for selected phytoplankton taxa

The proportion of C>T changes on the first ten base pairs of the DNA reads are given for six intervals (0-11.2 ka BP, 11.7-18.7 ka BP, 20.0-47.0 ka BP, 52.7-87.8 ka BP, 94.0-111.1 ka BP, 120.0-123.8 ka BP). The number of reads used for the analyses are given after the taxon name. “_noise” indicates the ratio of all other base pair changes at the first until the tenth position of the DNA read.

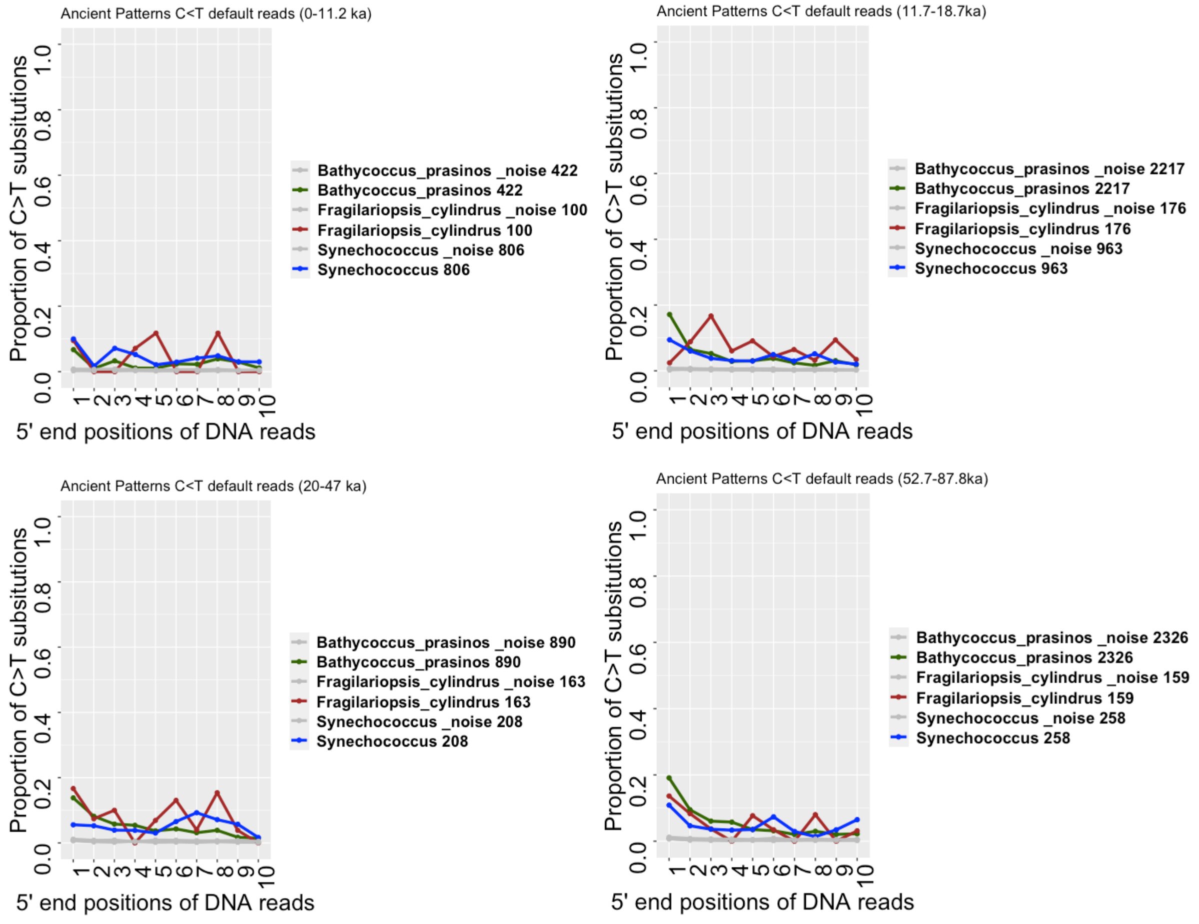

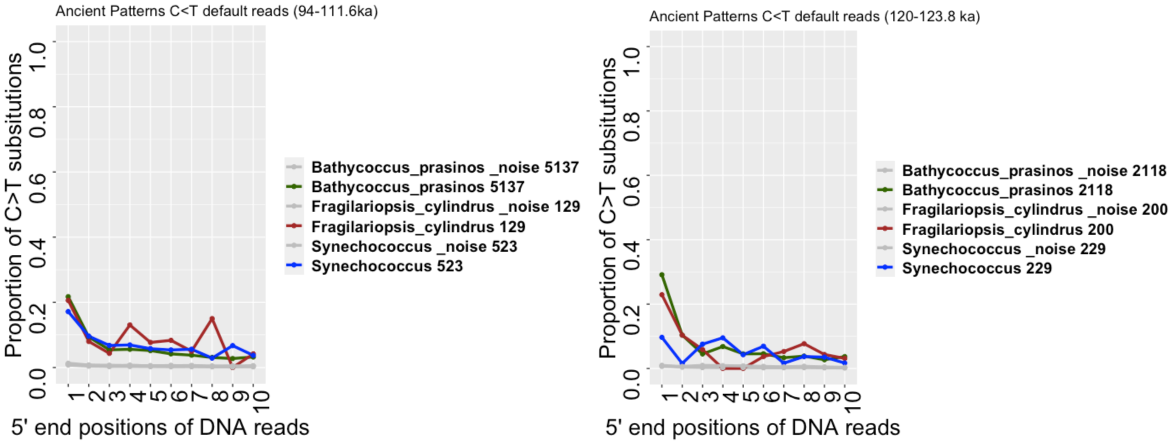


Figure S8A: Default reads for selected phytoplankton taxa.

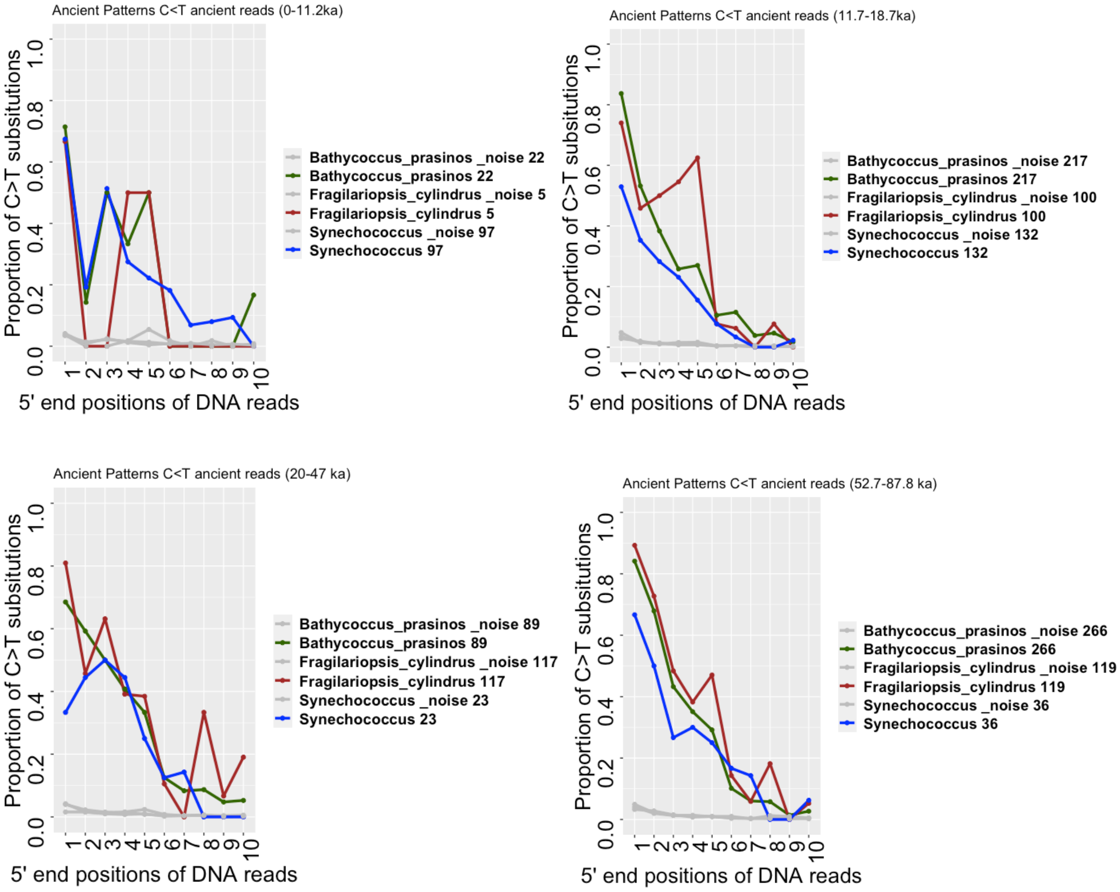

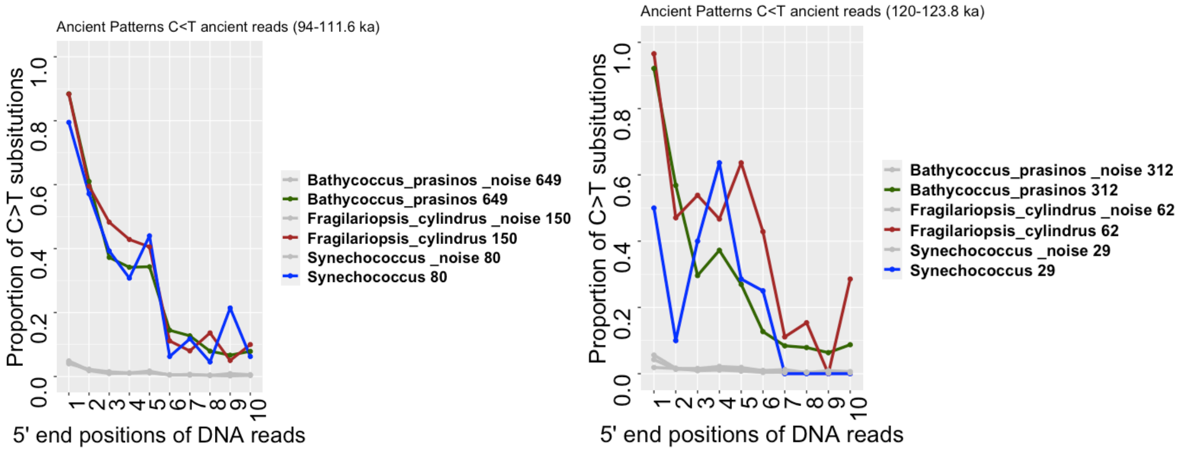


Figure S8B: Ancient reads for selected phytoplankton taxa.

**Damage pattern analysis for selected zooplankton taxa**

The proportion of C>T changes on the first ten base pairs of the DNA reads are given for six intervals (0-11.2 ka BP, 11.7-18.7 ka BP, 20.0-47.0 ka BP, 52.7-87.8 ka BP, 94.0-111.1 ka BP, 120.0-123.8 ka BP). The number of reads used for the analyses are given after the taxon name. “_noise” indicates the ratio of all other base pair changes at the first until the tenth position of the DNA read.

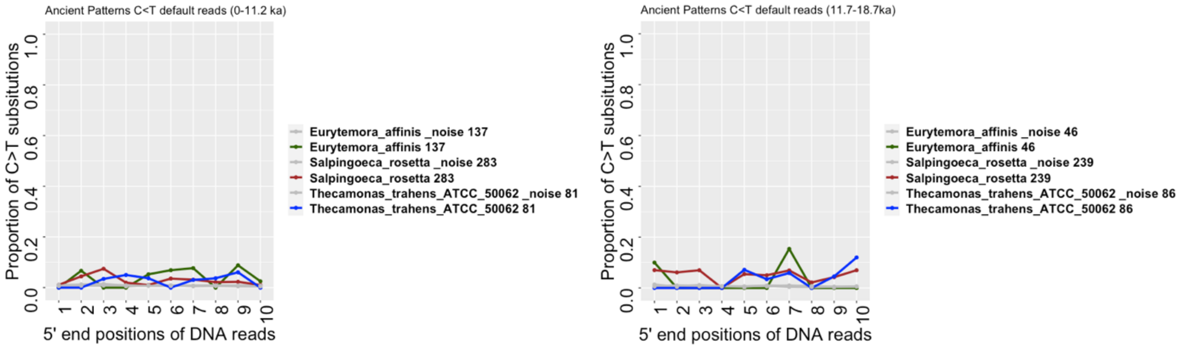

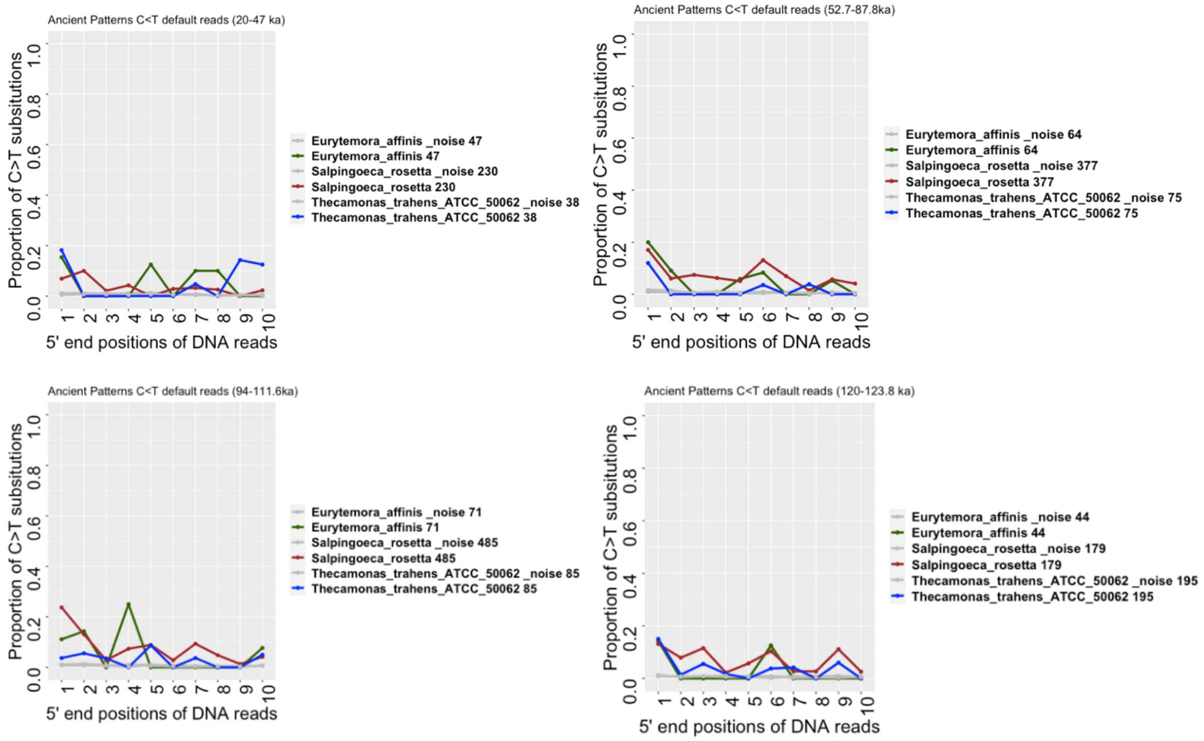


Figure S9A: Default reads for selected zooplankton taxa.

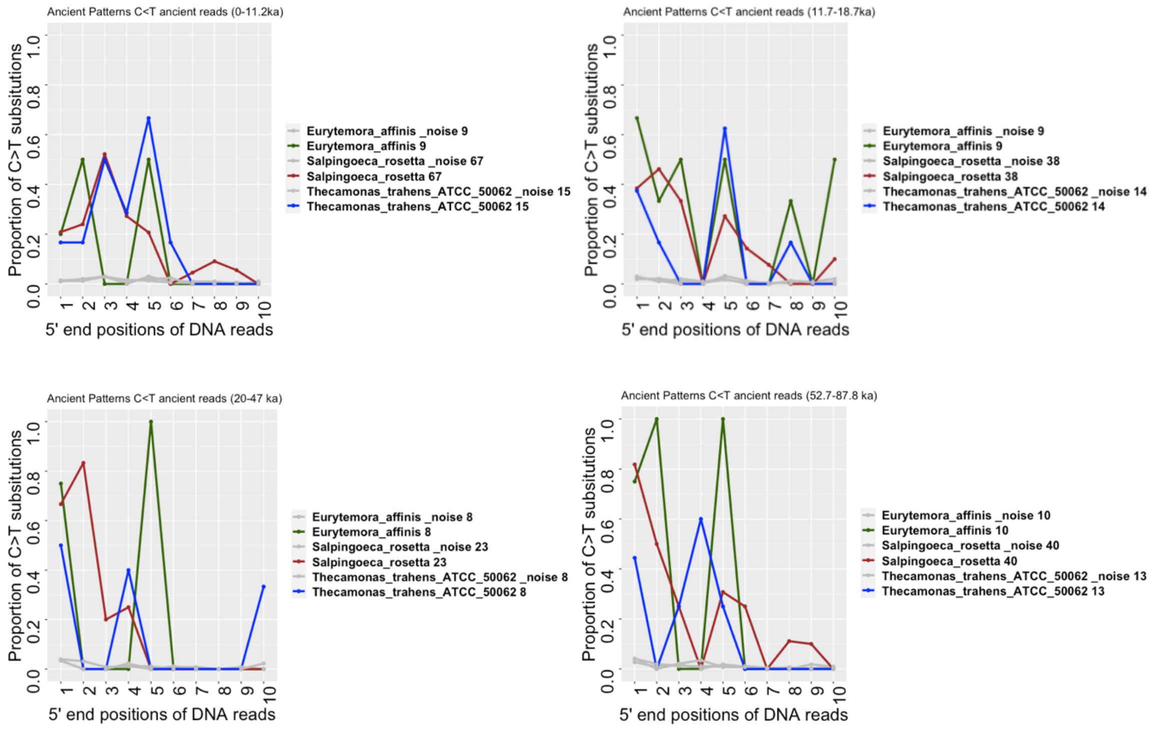

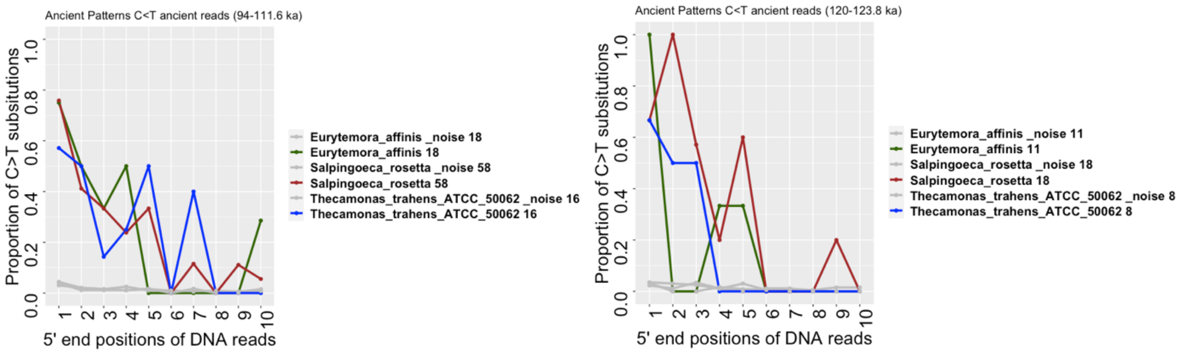


Figure S9B: Ancient reads for selected zooplankton taxa.

## General characterization of the phytoplanktonic community from the shotgun metagenomic approach

Phytoplankton groups divide in phototrophic protists (49.5%), chlorophyte algae (19.8%) and phototrophic bacteria (30.6%). Phototrophic protists comprise of micro-sized diatoms (31.3%) and nano-sized algae including haptophytic Phaeocystaceae and Noelaerhabdaceae (8.1%), and algae of the other representatives of the SAR complex (excluding diatoms), which account for totally for 4.5% and include Triparmaceae (3.1%), Monodopsidaceae (1.4%) and dinoflagellates (0.6%). Chlorophyte algae comprise pico-sized families, namely Bathycoccaceae (10.8%), Mamiellaceae (2.9 %) and Chloropicaceae (2.5%). Phototrophic bacteria comprise 23 families with Synechococcaceae (34.9%), Nostocaceae (14.8%) and Chlorobiaceae (9.8%) being the most dominant. The temporal abundance pattern of all 66 detected phytoplankton families is shown in Figure S10.

Eight highly abundant zooplankton families were detected (Calanidae, Metridinidae, Temoridae, Apusomonadidae, Salpigoecidae, Sphaerozoidae, Parameciidae, Vahlkampfiidae). They divide into heterotrophic protists (85.5%), crustaceous (11%) and gelatinous zooplankton (3.5%). Among the heterotrophic protists Sphaerozoidae (39.6%), Salpingoecidae (9.6%) and Apusomonadidae (6.4%) predominate. Crustaceous zooplankton, typically grazing on diatoms, comprise five families with Temoridae (4.7%) and Calanidae (2.5%) being dominant. The lowest abundance was detected for gelatinous zooplankton (6 families) (for read counts before and after resampling see: Supplementary Data 2 or https://doi.org/10.5281/zenodo.10064386).

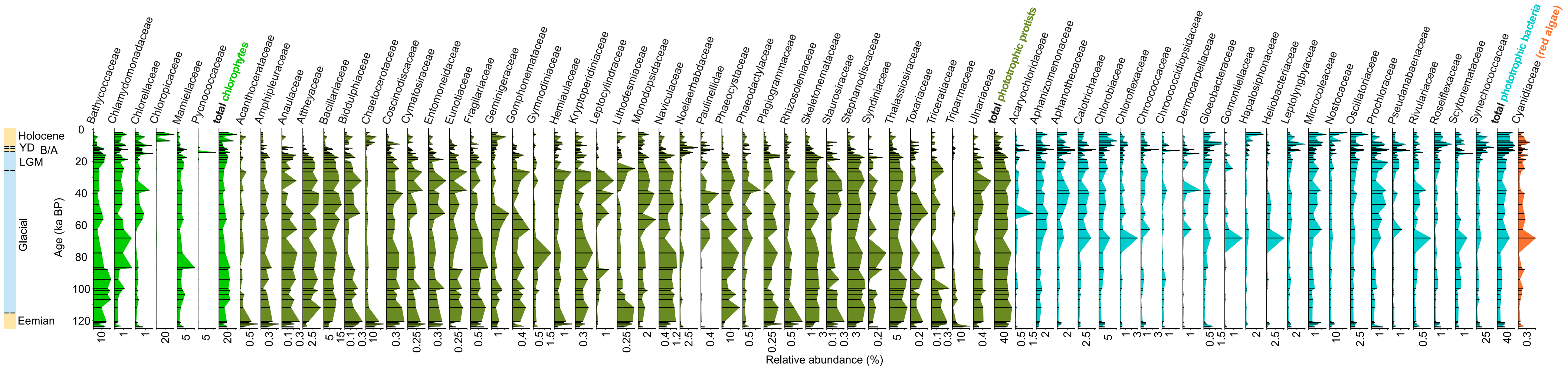


Figure S10: All phytoplankton families detected by the shotgun approach plotted versus age. Relative abundance is calculated from all phytoplankton reads of the respective sample after resampling. Warm periods (Holocene, Bølling-Allerød - B/A, Eemian) and cool periods (Younger Dryas (YD), Last Glacial Maximum (LGM), and the last glacial period) are indicated on the left.

**General characterization of the diatom community from the diatom amplicon-sequencing approach**

Centric diatoms in the resampled dataset account for 83% from all reads and pennate diatoms make up 17%. The dominant centric diatom families detected by amplicon-sequencing are Thalassiosiraceae (51% from total, 199 ASVs, 7 genera), Chaetocerotaceae (21%, 102 ASVs, 1 genus) and Attheyaceae (8.6%, 42 ASVs, 1 genus) (Supplementary Table S2; for resampled dataset see Supplementary Data 6 or https://doi.org/10.5281/zenodo.10064386). Predominant pennate diatom families include Bacillariaceae (7%, 66 ASVs, 5 genera), Naviculaceae (2.8%, 16 ASV, 3 genera) and Amphipleuraceae (3%, 14 ASVs, 1 genus).

Table S2: Number of Amplicon Sequence Variants (ASVs) assigned to diatom families and genera. Total number of ASVs per family and genus prior to resampling is given.

| family | Number of ASVs | genus | Number of ASVs |
|---|---|---|---|
| Achnanthidiaceae | 3 | *Achnanthidium* | 3 |
| Amphipleuraceae | 14 | *Halamphora* | 14 |
| Attheyaceae | 42 | *Attheya* | 42 |
| Bacillariaceae | 66 | *Bacillaria* | 17 |
| | | *Cylindrotheca* | 12 |
| | | *Nitzschia* | 29 |
| | | *Psammodictyon* | 1 |
| | | *Pseudo-nitzschia* | 5 |
| Chaetocerotaceae | 102 | *Chaetoceros* | 102 |
| Cymatosiraceae | 2 | *Arcocellulus* | 1 |
| Entomoneidaceae | 6 | *Entomoneis* | 6 |
| Fragilariaceae | 9 | *Fragilaria* | 6 |
| | | *Grammonema* | 1 |
| | | *Synedra* | 1 |
| | | *Synedropsis* | 1 |
| Gomphonemataceae | 3 | *Gomphonema* | 3 |
| Hemiaulaceae | 2 | *Hemiaulus* | 2 |
| Hemidiscaceae | 12 | *Actinocyclus* | 12 |
| Leptocylindraceae | 1 | *Leptocylindrus* | 1 |
| Melosiraceae | 1 | *Melosira* | 1 |
| Naviculaceae | 16 | *Haslea* | 8 |
| | | *Navicula* | 5 |
| | | *Pseudogomphonema* | 2 |

| | | | |
|---|---|---|---|
| Odontellaceae | 2 | *Odontella* | 2 |
| Rhabdonemataceae | 1 | *Rhabdonema* | 1 |
| Rhizosoleniaceae | 1 | *Rhizosolenia* | 1 |
| Stephanodiscaceae | 1 | *Stephanodiscus* | 1 |
| Thalassionemataceae | 1 | *Thalassionema* | 1 |
| Thalassiosiraceae | 199 | *Bacterosira* | 25 |
| | | *Conticribra* | 1 |
| | | *Detonula* | 1 |
| | | *Minidiscus* | 2 |
| | | *Porosira* | 56 |
| | | *Shionodiscus* | 1 |
| | | *Thalassiosira* | 108 |

**PCR Replicate similarity in diatom amplicon-sequencing approach**

To estimate the similarity between amplicon-sequencing replicates, a NMDS analysis was performed on the amplicon-sequencing dataset using the function *metaMDS*. Presence/absence data on the level of scientific name (best match with the database, independent from the taxonomic resolution) were used as input. Generally, replicates of the same sample show a similar diatom community composition, with glacial replicates being more similar to each other than interglacial replicates. In contrast, replication of samples taken at climate transition phases (e.g., at 120.38 ka BP: last Eemian sample before glacial period; 16.09 ka BP: last glacial sample before Bølling-Allerød and climate rebound) results in less similar communities between replicates (Fig. S11).

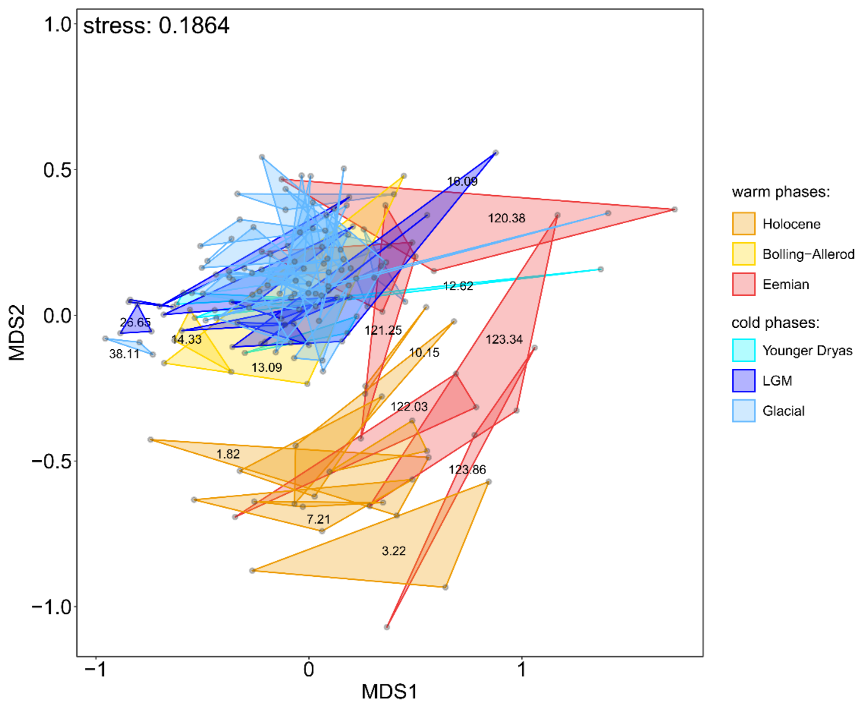


Figure S11: Non-metric multidimensional scaling (NMDS) of the diatom amplicon-sequencing replicates. Replicates of the same sample form a triangle. Samples are color coded according to the main climatic periods; LGM = Last Glacial Maximum.

## References


1. Riethdorf JR, Nürnberg D, Max L, Tiedemann R, Gorbarenko SA, Malakhov MI. Millennial-scale variability of marine productivity and terrigenous matter supply in the western Bering Sea over the past 180 kyr. Clim Past. 2013;9:1345–1373.

2. Max L, Riethdorf JR, Tiedemann R, Smirnova M, Lembke-Jene L, Fahl K, et al. Sea surface temperature variability and sea-ice extent in the subarctic northwest Pacific during the past 15,000 years. Paleoceanography. 2012;27.3.

3. Stuiver M, Reimer PJ. Extended $^{14}C$ data base and revised Calib 3.0 $^{14}C$ age calibration program. Radiocarbon. 1993;35:215–230.

4. Reimer P, Ballie M, Bard E, Bayliss A, Beck J, Blackwell P, et al. IntCal09 and Marine09 radiocarbon age calibration curves. Radiocarbon. 2009;51:1111–1150.

5. Wang YJ, Cheng H, Edwards RL, An ZS, Wu JY, Shen CC, et al. A high-resolution absolute-dated late pleistocene monsoon record from Hulu Cave, China. Science. 2001;294:2345–2348.

6. Wang Y, Cheng H, Edwards RL, Kong X, Shao X, Chen S, et al. Millennial- and orbital-scale changes in the East Asian monsoon over the past 224,000 years. Nature. 2008;451:1090–1093.

7. Rasmussen SO, Andersen KK, Svensson AM, Steffensen JP, Vinther BM, Clausen

HB, et al. A new Greenland ice core chronology for the last glacial termination. J Geophys Res Atmos. 2006;111:D16.

8. Channell JET, Xuan C, Hodell DA. Stacking paleointensity and oxygen isotope data for the last 1.5 Myr (PISO-1500). Earth Planet Sci Lett. 2009;283:14–23.

9. Lisiecki LE, Raymo ME. A Pliocene-Pleistocene stack of 57 globally distributed benthic δ 18O records. Paleoceanography. 2005;20:PA1003.

10. Paillard D, Labeyrie L, Yiou P, Paillard D, Labeyrie L, Yiou P, et al. Macintosh Program performs time-series analysis To cite this version : HAL Id : hal-03334909 Macintosh Program Performs Time-Series Analysis. 1996;77:379.

11. Stoof-Leichsenring KR, Epp LS, Trauth MH, Tiedemann R. Hidden diversity in diatoms of Kenyan Lake Naivasha: A genetic approach detects temporal variation. Mol Ecol. 2012;21:1918–1930.

12. Hübler R, Key FM, Warinner C, Bos KI, Krause J, Herbig A. HOPS: automated detection and authentication of pathogen DNA in archaeological remains. Genome Biol. 2019; 20:280.

13. Boyer F, Mercier C, Bonin A, Le Bras Y, Taberlet P, Coissac E. OBITOOLS : a UNIX - inspired software package for DNA metabarcoding. 2016;16:176–182.

14. Lepot M, Aubin JB, Clemens FHLR. Interpolation in time series: An introductive overview of existing methods, their performance criteria and uncertainty assessment. 2017;9:796.
